# Carotenoids in Health as Studied by Omics-Related Endpoints

**DOI:** 10.1016/j.advnut.2023.09.002

**Published:** 2023-09-09

**Authors:** Torsten Bohn, Emilio Balbuena, Hande Ulus, Mohammed Iddir, Genan Wang, Nathan Crook, Abdulkerim Eroglu

**Affiliations:** 1 Nutrition and Health Research Group, Department of Precision Health, Luxembourg Institute of Health, Strassen, Luxembourg; 2 Department of Molecular and Structural Biochemistry, College of Agriculture and Life Sciences, North Carolina State University, Raleigh, NC, United States; 3 Plants for Human Health Institute, North Carolina Research Campus, North Carolina State University, Kannapolis, NC, United States; 4 Department of Chemical and Biomolecular Engineering, College of Engineering, North Carolina State University, Raleigh, NC, United States

**Keywords:** health, inflammation, β-carotene, lycopene, lutein, oxidative stress, metabolites, LC-MS-MS, exposome, transcription factors, cellular compartments, body tissues

## Abstract

Carotenoids have been associated with risk reduction for several chronic diseases, including the association of their dietary intake/circulating levels with reduced incidence of obesity, type 2 diabetes, certain types of cancer, and even lower total mortality. In addition to some carotenoids constituting vitamin A precursors, they are implicated in potential antioxidant effects and pathways related to inflammation and oxidative stress, including transcription factors such as nuclear factor κB and nuclear factor erythroid 2-related factor 2. Carotenoids and metabolites may also interact with nuclear receptors, mainly retinoic acid receptor/retinoid X receptor and peroxisome proliferator-activated receptors, which play a role in the immune system and cellular differentiation. Therefore, a large number of downstream targets are likely influenced by carotenoids, including but not limited to genes and proteins implicated in oxidative stress and inflammation, antioxidation, and cellular differentiation processes. Furthermore, recent studies also propose an association between carotenoid intake and gut microbiota. While all these endpoints could be individually assessed, a more complete/integrative way to determine a multitude of health-related aspects of carotenoids includes (multi)omics–related techniques, especially transcriptomics, proteomics, lipidomics, and metabolomics, as well as metagenomics, measured in a variety of biospecimens including plasma, urine, stool, white blood cells, or other tissue cellular extracts. In this review, we highlight the use of omics technologies to assess health-related effects of carotenoids in mammalian organisms and models.

**Statement of significance**

This article emphasizes the potential of various omics techniques in carotenoid research targeting human health. Such an overview, pointing out limitations and gaps, and perspectives for carotenoid-related omics work is currently underappreciated. However, such orchestrated research is much needed to move carotenoid-based research forward.

## Introduction

Carotenoids are typically colorful C40 tetra-terpenoid pigments produced by a variety of plants, bacteria, and fungi. Although over 1100 carotenoids have been reported to exist in nature [1], only a handful play a role in the human diet [2]. Despite not generally constituting essential nutrients, these dietary carotenoids may play important roles in human metabolism and health. Some carotenoids can act as precursors to vitamin A, resulting in the metabolism to shorter apo-carotenoids such as retinol, e.g., out of β-carotene and β-cryptoxanthin by the action of β-carotene oxygenase 1 (BCO1) [3]. Others, predominantly lutein and zeaxanthin, have been reported to aid in the prevention against age-related macular degeneration (AMD), the leading cause of vision loss in the elderly [4]. Though no clear causal relations can be inferred, carotenoid dietary intake has been associated in prospective studies with the prevention of a number of chronic diseases, including type 2 diabetes [5] and cardiovascular disease (CVD) [6]. The health benefits of carotenoids have originally been attributed to their antioxidant activity, acting as potential quenchers of reactive oxygen species (ROS) [7]. However, more recently it has been emphasized that their interactions with transcription factors may play a more important role, such as with nuclear factor erythroid 2-related factor 2 (Nrf2) and nuclear factor kappa B (NF-κB) and also nuclear retinoid receptors such as retinoic acid receptors (RARs)/retinoid X receptor (RXR), involved in cellular differentiation [8].

Despite their associations with health-related outcomes, it is often unclear whether the benefits derive from carotenoids alone or their consumption in a whole-food context, namely fruits and vegetables, which include other beneficial compounds, such as dietary fiber. Consequently, the biological disentanglement between the mechanistic action of carotenoids and health-beneficial effects from other micronutrients or nonnutrients are an important aspect of research. However, although additional insights into the action of carotenoids have been revealed in the last decades, many aspects still need to be better understood. Omics-based techniques appear most suitable for this task due to the potentially complex interactions of carotenoids with transcription factors and nuclear factors and thus with a large number of downstream genes and expressed proteins that can produce many changes in the body. In this regard, it is hoped that further insights from omics-based studies can shed light on the complex relationship between carotenoid intake and their potential health benefits or even arbitrary aspects, as high concentrations of at least β-carotene from supplemental doses have been reported to be detrimental to some populations, such as smokers [9], increasing the risk of lung cancer.

Various omics techniques have been applied in carotenoid research, including proteomics [10,11], transcriptomics [12,13], metabolomics [14,15], and lipidomics, which includes measuring carotenoid metabolites [16,17], as well as genomics [18,19]. Furthermore, related to gut microbiota effects, metagenomics has been applied, i.e., the detection of nucleotide sequences and their functions isolated from all microorganisms in a sample. However, predominantly the marker gene approach (16S rRNA) has been employed [20,21] rather than whole metagenome sequencing or shotgun metagenome sequencing. However, research is hampered by the need for expensive equipment, such as mass spectrometers (MS) for metabolomics, next-generation sequencers for metaomics, and nano-liquid chromatography (LC) and MS for proteomics, and the dearth of specialists to prepare samples and analyze and interpret the resulting sophisticated datasets. The same applies to multiomics approaches, which would require a joined, orchestrated investigation by a large multidisciplinary team.

A few examples can be given to exemplify the usefulness of omics studies in carotenoid research. For instance, an untargeted proteomics approach was applied to determine the postprandial effect of feeding supraphysiological concentrations of various carotenoids and retinol to Mongolian gerbils [11], a model suitable to study carotenoid metabolism. Liver and adipose tissue (by a 2-dimensional difference gel electrophoresis [2D-DIGE] approach) and plasma (LC-MS approach) expression of proteins were examined, showing that some proteins were differently regulated compared with vehicle only. Another example is that Eroglu et al. [10] demonstrated that MS-based proteomics could be used as a proxy to assess carotenoid status in populations. Such studies can reveal novel insights into broader effects of carotenoids, even acute ones, on the overall metabolism and reveal differential effects between various (apo-) carotenoids. Another study by Peng et al. [14] focused on targeted metabolomics relating circulating carotenoids and plasma metabolites in a nested case-control study related to breast cancer, revealing metabolites by high performance liquid chromatography – tandem mass spectrometry (LC-MS/MS) that were associated with main circulating carotenoids. In addition, carotenoid-related signatures such as that of β-carotene were related to a lower risk of breast cancer, emphasizing the preventive effects of carotenoids, their related biological signatures, and potential mechanisms.

Multiomics, due to their high degree of complexity and the need to align the various omics outcomes, have much less been applied in carotenoid research. However, this cross-cutting experimental approach combining e.g., metabolomics and transcriptomics has started to receive some attention [22]. Thus, such omics approaches may pave the way toward novel biomarkers and an improved understanding of the biological activity of carotenoids. In this review, which is directed especially to interested nutritionists and researchers in the carotenoid field that have thus far not employed omics techniques in their research, we aim to report on the state-of-the-art employment of omics techniques to reveal insights into the relationship between carotenoid intake and health outcomes, summarizing the main findings obtained in relevant *in vitro*, animal, and human studies, as well as emphasizing gaps of knowledge and technological shortcomings.

## Relevance for relating carotenoid intake and status to health-relevant outcomes and overview of omics-techniques

The frequently consumed carotenoids in the diet include β-carotene, lycopene, lutein, zeaxanthin, β-cryptoxanthin, and α-carotene, though phytoene and phytofluene are also taken in at similar amounts from the normal diet, and likely also violaxanthin and neoxanthin [2,23]. Though the daily intake is in the mg range (up to approximately 20 mg/d), these constituents are the most abundant liposoluble secondary plant compounds in the plasma, with concentrations of up to several μmol/L [2]. Following their dietary intake, carotenoids are either absorbed intact or cleaved into apo-carotenoids. The latter happens either by central cleavage into retinoids, by BCO1, cleaving preferably provitamin A carotenoids but also lycopene, or eccentrically by BCO2, which cleaves rather lutein and lycopene (i.e., non-provitamin A carotenoids) and even other apo-carotenals [24]. BCO2 cleavage happens in the mitochondria and results in the production of a variety of apo-carotenals [25]. Further glucuronidation of these products in order to increase polarity for excretion via the kidney has been reported, as reviewed previously [26].

**Figure 1:**
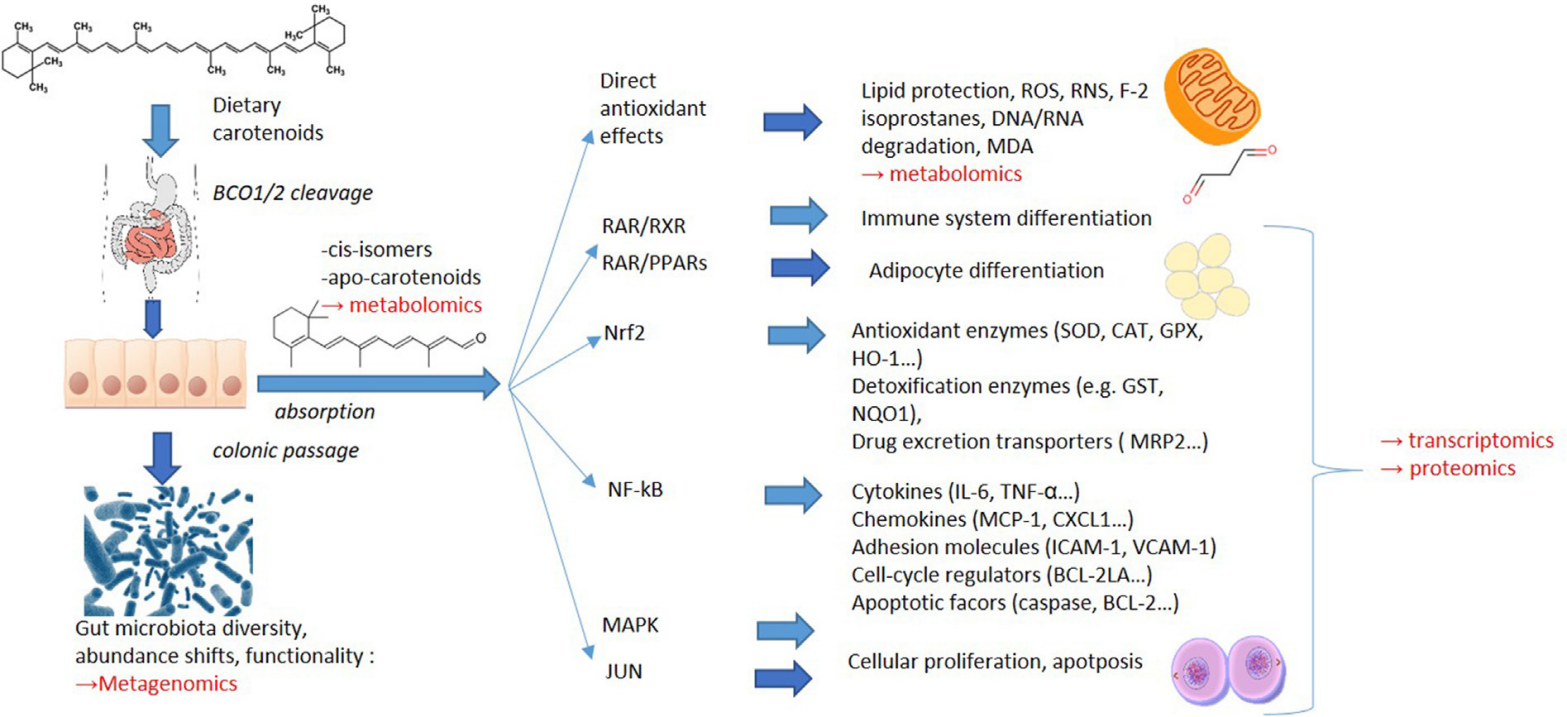
Overview of metabolically measurable endpoints that could be plausibly related to carotenoids – from influences on gene transcription to downstream proteins and metabolites to further potential biological targets. BCL, B cell lymphoma; BCO, β-carotene oxygenase; CAT, catalase; CXCL, chemokine (C-X-C motif) ligand; GPX, glutathione peroxidase; GST, glutathione-S-transferase; ICAM, intracellular adhesion molecule; IL, interleukin; MDA, malondialdehyde; NQO, NADPH quinone oxidoreductase; MCP, monocyte chemoattractant protein; MRP2, multiple drug resistance protein 2; RNS, reactive nitrogen species; ROS, reactive oxygen species; SOD, superoxide dismutase; TNF-α, tumor necrosis factor alpha; VCAM, vascular cell adhesion molecule.

Carotenoids are intertwined with a large number of biological pathways and endpoints in the human body that omics techniques may target. These include (Figure 1):

1) Interactions, possibly following cleavage by BCO1/2, with transcription factors related to NF-κB [8,27], a “master-switch” involved in inflammation [28], and therefore further downstream genes associated with the expression of a large number of cytokines [29] such as tumor necrosis factor alpha (TNF-α), interleukins (e.g., IL-6, IL8, IL-1β), chemokines (e.g., monocyte chemo-attractant protein 1 [MCP-1], chemokine (C-X-C motif) ligand 1 [CXCL1]), adhesion molecules (intercellular adhesion molecule 1 [ICAM1], vascular cell adhesion molecule 1 [VCAM1]), cell-cycle regulators (e.g., B-cell lymphoma-extra large [BCL-xL], BCL-2, BCL-2L1), and apoptotic factors (e.g., caspase);
2) Similarly, following partial cleavage into apo-carotenoids, which could impact the transcription factor Nrf2 [30,31], a “master switch” for the bodies’ own antioxidant system [32], regulating the expression of antioxidant enzymes (such as superoxide dismutase [SOD], glutathione peroxidase [GPx], catalase [CAT], heme-oxygenase 1 [HO-1]), detoxification enzymes (e.g., glutathione S-transferases [GSTs], NADPH quinone oxidoreductase [NQO1]), and drug excretion transporters (e.g., multiple drug resistance protein 2 [MRP2]), among others [33];
3) Interactions with nuclear receptors, as vitamin-A-active retinoids, at least retinoic acid, interact with RAR, which, together with RXR or PPARs [26, 34], impinge on >500 downstream gene targets [35] that regulate a large number of cellular differentiation pathways related to apoptosis, cellular division, development of the immune system, embryonic development, adipocyte differentiation, and many more [36,37]. Interactions of other metabolites, such as 9-cis-retinoic acid on RXR, have also been reported [38].
4) Additional interactions with further transcription factors, such as mitogen-activated protein kinase (MAPK) [39], also involved in cellular differentiation and proliferation, among others, or JUN [8], relevant for cellular differentiation and apoptosis;
5) Likely interactions with ROS, as carotenoids may function as antioxidants, scavenging ROS, aiding in the prevention of lipid peroxidation [7], and perhaps even reducing reactive nitrogen species [40]. This may impact a variety of lipophilic molecules, such as preventing lipid oxidation and formation of reactive products such as malondialdehyde (MDA) [41], their effect on F2-isoprostanes [42] and prostaglandins [43], as well as the measurement of the formed apo-carotenoids themselves [44], but also the prevention of DNA/RNA breakdown products such as 8-hydroxy-2′-deoxyguanosine and perhaps improving plasma antioxidant activity [7];
6) More recently, the potential impact on the gut microbiome has been discussed [45]. Carotenoids may influence gut microbiota via several pathways. This could be due to reducing the pro-oxidant potential in the gut, effects on immunoglobulin A (IgA) that are related to vitamin A activity (for some carotenoids) and potential bactericidal effects, as reviewed previously [45,46]. Thus, characterizing biological activities by metagenomics is another potential route to determine health-beneficial aspects of carotenoids.

Carotenoid-related pathways could influence health via a broad array of mechanisms, and thus omics or multiomics signatures may detect these manifold influences on the human body, from gene transcription to protein expression and furthermore to downstream metabolic compounds, rather than focusing on only a single endpoint. This is important, especially in light of the fact that there are no clear and accepted individual metabolic endpoints that relate carotenoid intake or status with chronic diseases, except for macular pigment optical density for AMD [4]. Another advantage of targeting several endpoints at a time is to distinguish carotenoid-related endpoints from those potential confounders, such as when carotenoids are given within fruits and vegetables, which are expected to also trigger health-beneficial pathways upon their consumption [47].

Transcriptomics, following mRNA isolation, can be a powerful tool for detecting a broad number of expressed genes – up to several thousand in chip-based microarrays [48,49] or whole transcriptome RNA sequencing [50]. However, targeted approaches, such as by qPCR, have been more common due to a lower cost and number of samples needed compared to untargeted analyses [12,51]. A challenge of transcriptomics may be that the time of altered gene expression may be limited [52] and could be missed.

Proteomics detects differentially regulated proteins within a specific organ, tissue, or plasma, although proteins from subcellular fractions (such as the cytosol, nucleus, or cell membrane [53]) may also be harvested. As proteomics is downstream from transcriptomics, its results may better represent the bioactivity of carotenoids because posttranscriptional modifications and time-dependent expression of mRNA may result in further biological changes not captured at the transcriptomic level. Approaches can be targeted [54] or untargeted [55,56], applying, e.g. gel-based (such as 2D-DIGE) or gel-free approaches such as by nano-LC [57]. Also, both native and denatured protein techniques [58] have been described at the level of separation, such as by gels, with native proteomics allowing the distinction between different proteoforms, i.e., variations or different molecular forms of proteins [59]; however, the technique has, to our knowledge, not yet employed for carotenoid and health related proteomics.

Metabolomics, including lipidomics, assesses downstream effects by measuring a large number of metabolites in plasma, tissues, or organelles [60], employing LC or gas chromatography [61] coupled to mass spectrometry (GC-MS) [61]. Also, nuclear magnetic resonance (NMR) techniques have been used to determine metabolite signatures resulting from various dietary exposures but may be limited by sensitivity issues [62]. Both targeted and untargeted approaches, including the detection and quantification of carotenoids, have been reported, as reviewed previously, with the aim to detect carotenoids and metabolites within lipophilic fractions [15].

Finally, metagenomics may constitute a novel approach of assessing the action of carotenoids on gut microbiota. The majority of carotenoids are not absorbed and will reach the colon [45]. Carotenoids have been associated with altered gut microbiota in intervention studies [21] assessing relative abundance of bacteria by either 16S rRNA sequencing at the genus level or shotgun metagenomics at the species or strain level to obtain an overview on the functional potential of microbial communities [63]. Thus, metagenomics constitutes a novel and complementary approach to study the bioactivity of carotenoids [45,63].

A final important difference exists between targeted compared with non-targeted methods, and both have been employed in the carotenoid field. Targeted approaches have the advantage of an a priori hypothesis that can be validated, and the focus on fewer targets allows for a higher statistical power to detect changes. Untargeted approaches may offer broader insights without any a priori hypothesis. However, statistical interpretation, including post hoc adjustments for multiple techniques such as by Benjamini-Hochberg [64], due to the typically much larger number of endpoints, remains more challenging. Such studies usually require a larger number of humans or animals to improve statistical power.

## Effect of Carotenoids as Assessed by Transcriptomics

### Introduction

Transcriptomics refers to measuring RNA expression in an entire biological entity, typically a cell population. In a stricter sense, it refers to assessing the complete set of RNA or transcripts [65]. Distinctions are made between targeted transcriptomics, in which only a specific type of mRNA expression is measured, typically by qPCR, and untargeted or global transcriptomics, assessing the entity of expressed RNA by microarrays or RNA sequencing (RNA-seq) [57]. RNA-seq can also be targeted to sequence-specific transcripts rather than the whole transcriptome. Many studies apply a combination of these techniques, for instance, confirming findings obtained by RNA-seq with targeted qPCR in a second step. The choice of method depends on the underlying research question: if only a few selected mRNA targets are chosen, qRT-PCR is the method of choice. If a larger number of endpoints are targeted, and the organism has been sequenced, microarrays may be the method of choice, whereas for a hypothesis-free approach, RNA-seq should be employed, though it comes at higher costs and the need for large sample sizes and a more complex statistical evaluation.

Transcriptomics encompasses various types of RNA, including mRNA but also noncoding RNAs (ncRNAs) such as microRNAs (miRNAs), long ncRNAs (lncRNAs) [66,67], and rRNA. Transcriptomics investigates transcription and expression levels, locations, trafficking, degradation, and information about the related genes, splicing patterns, and posttranscriptional modifications [65].

An interesting and promising area is ncRNAs, which are present at lower concentrations (compared to all RNA in a typical cell) than the already low 2% to 3% mRNA (the majority being rRNA and tRNA) [68]. To our knowledge, ncRNAs, such as tRNAs, rRNAs, miRNAs, small interfering RNAs, and lncRNAs, have scarcely been investigated in relation to carotenoid research; however, there has been some interest in this domain recently. For instance, during the COVID-19 crisis, lncRNA (i.e., lncRNA idiopathic pulmonary fibrosis [ITBF]) was found at elevated levels and was associated with the integrin subunit beta like 1 (*ITGBL1*) gene in pulmonary fibrogenesis responsible for fibroblast differentiation and, thus, pulmonary fibrosis. Interestingly, in the study by Chen et al. [69], astaxanthin hampered fibroblast proliferation through lnclTPF and mitochondria-related signaling pathways. Such studies, including those regarding ncRNAs, are believed to harbor novel potential in carotenoid-related research, as ncRNAs are widely expressed, interact with a number of genes, and have, to a large extent, been overlooked in research thus far.

A potential challenge in transcriptomics is that the expression level may change swiftly with time, i.e., even without intervention, due to circadian rhythm [70], which may be relevant for carotenoid related activity, as suggested for retinoid receptors [71]. Thus, even time series of RNA expression levels may be followed to obtain a more clear picture of the activation of certain pathways [72], a technique that may be relevant for carotenoids, considering certain of their downstream effects may vary according to circadian activities such as oxidative stress [73] or the immune system [74], although this is methodologically more challenging.

Biological material of interest may vary widely, but due to relatively high concentrations of carotenoids targeting these tissues or due to availability, white adipose tissue, liver, or plasma are frequently employed targets [2,11]. For RNA extraction purposes, commercial kits are typically used, which also allow the isolation of miRNA. For further information, the reader is referred to more in-depth reviews [75,76].

### Methodological overview

#### qRT-PCR

This method is employed for preselected target sequences. In carotenoid research, this may entail a large variety of genes, such as those related to transporters of carotenoids (e.g., scavenger receptor class B type 1 (*SCARB1*) or genes activated by vitamin A, such as retinol binding protein 4 (*RBP4*) and *RARβ* [77]. In multiplex PCR, more than one target sequence can be detected at the same time. During qRT-PCR, RNA is reverse transcribed in a first step into cDNA using reverse transcriptase. The resulting cDNA is then amplified [78]. DNA is quantified within qRT-PCR by fluorescence binding dyes such as SYBR green [79]. For quality assurance, the MIQE guidelines may be followed [79,80].

#### Untargeted transcriptomics by microarray assays

This technique is employed when targeting predefined sequences, which requires previous sequencing of the organism. Single nucleotide polymorphism analysis has often been carried out by microarray assays, including carotenoid research [18,81]. cDNA is first synthesized and amplified, similar to RT-PCR, and the DNA is labeled with fluorescence dyes such as cyanine 5-aminoallyl cytidine-5'-triphosphate (5-CTP) or cyanine 3-CTP for later detection [57]. Typically, a reference standard is analyzed in parallel and labeled with one of the dyes, whereas the sample is labeled with the other. Then, cDNA is hybridized to a microarray chip to which target cDNA fragments can bind, given that their complementary sequences are present on the chip. Microarrays typically contain several thousand nucleic acid targets. The advantage is typically the price, being more affordable than RNA-seq and still offering the possibility to detect many targets. However, RNA-seq is generally applied for more explorative approaches, i.e., when specific gene targets are not known. Disadvantages include that splice variants are typically undetected, and the hybridization may be nonspecific. Microarray chips have been developed for humans, mice, pigs, and rabbits but do not appear to exist for gerbils, which are a good model for carotenoid metabolism, even though Mongolian gerbil miRNA was studied based on microarray technology [82]. For further information, the reader is referred to more comprehensive reviews [83,84].

#### Untargeted transcriptomics by RNA-seq

RNA-seq does not rely on previously defined sequences/targets and can thus be employed for hypothesis-free approaches across the entire transcriptome, including ncRNAs. Alternative splice sites can likewise be investigated. In carotenoid research, this method may best be used for animal models for which microarrays may not be available, such as Mongolian gerbils.

The main steps of RNA sequencing are *1*) template preparation (i.e., isolation of nucleic acid); *2*) library preparation (repairing ends, ligation of adapters, optional amplification by PCR); and *3*) sequencing (preparation and reading of sequences) [85]. A typical read length of the sequences is 150 kb for eukaryotic cells, and a typical coverage/sequence depth or read (number of times a nucleotide has been read, average value) would be 30. For more in-depth information, the reader is referred to further literature [86,87].

In addition to PCR-based techniques, there are also PCR-free technologies. Compared to PCR-based approaches, PCR-free approaches can reduce library bias and gaps in the sequence [88]. For further information, the reader is referred to more in-depth reviews [85,88,89]. Targeted RNA-seq can also be utilized to sequence particular genes of interest, either through target capture (biotinylated probes bound to cDNA/RNA) or amplicon sequencing (gene-specific primers for cDNA targets) approaches [90].

### Recent applications

#### Targeted qPCR

A large number of studies, mostly on animals, have employed qPCR in carotenoid and health research. Several studies focused on lycopene due to its strong antioxidant properties *in vitro*. Data from qPCR showed that lycopene (2 μM for 24 h) downregulated proinflammatory cytokine (*Il-6*) and chemokine (*Mcp-1*) levels in both *ex vivo* mouse adipose tissue explants and human 3T3-L1 preadipocytes that were pretreated with 15 ng/mL TNF-α [91]. The same study revealed a lycopene-induced (2 μM in plasma for 6 wk) reduction of proinflammatory *Il-1β*, *Il-6*, and *Mcp-1* levels in the white adipose tissue of male C57BL/6J mice on a high-fat diet (35% kcal from fat) compared to the group without lycopene [91]. Similarly, lycopene (0.5–2 μM for 24 h) inhibited the lipopolysaccharide (LPS)-induced inflammatory response in RAW 264.7 macrophages and 3T3-L1 preadipocytes, primarily through inhibition of proinflammatory cytokine/chemokine (*Tnf-α*, *Il-6*, *Mcp-1*) levels as depicted by qPCR in both cell types [92]. When moving to *in vivo* studies, lycopene and tomato powder (10 mg/kg body weight (bw)/d for 12 wk) supplementation under high-fat (45% kcal from fat) conditions led to a reduction of proinflammatory cytokine/chemokine mRNA expression in the liver and epididymal adipose tissues of male C57BL/6J mice as measured by qPCR. Within the same study, genes involving hepatic lipid metabolism/steatosis (acetyl-CoA carboxylase alpha [*Acacα*]*,* fatty acid synthase [*Fasn*]*,* sterol regulatory element binding protein 1c [*Srebp-1c*]*)* and PPARγ activity were also reduced by lycopene and tomato powder administration [93]. These studies demonstrated that lycopene alone or with tomato powder could influence a large number of pathways associated with inflammation and adipose tissue metabolism.

As carotenoids may also accumulate in the liver, nonalcoholic fatty liver disease (NAFLD) has been the subject of some investigations. Astaxanthin administration (10, 30, 60 mg/kg bw every 2 d) to C57BL/6J mice on a high-fat diet (60% kcal from fat) for 10 wk alleviated NAFLD by reducing liver inflammation (TNF-α, IL-1β, inducible nitric oxide synthase [iNOS]) and promoting the fibroblast growth factor 21/PPARγ coactivator 1 alpha (FGF21/PGC-1α) pathway governing hepatic lipid metabolism [94]. In another study, Sprague-Dawley rats fed with a whole-food carotenoid source of spinach powder (2.5% or 5% in diet containing 20–24 or 50–53 μg total carotenoids (mixture of α-carotene, β-carotene, lutein, violaxanthin, and neoxanthin) alleviated NAFLD through the promotion of fatty acid (acyl-CoA dehydrogenase long chain [*Acadl*], *carnitine palmitoyltransferase II* [*Cpt2*]) and cholesterol, apolipoprotein A1 [*Apoa1*], low-density lipoprotein receptor [*Ldrl*], and APC regulator of WNT signaling pathway [*Apcg1*] metabolism via PPAR (*Pparα*, *Pparγ*, and *Pparδ*) overexpression [95].

Alternative treatments for various human health complications have been sought after and included carotenoid metabolites. A randomized, double-blinded, placebo-controlled clinical trial involving patients with coronary artery disease was conducted with targeted qPCR on participants’ peripheral blood mononuclear cells after administration of crocin, an apo-carotenoid derived from flowers of the crocus family and found in saffron extract, or aqueous saffron extracts (30 mg/d of either treatment for 8 wk). This trial found that crocin supplementation increased silent information regulator sirtuin 1 (*SIRT1*) and AMP activated protein kinase (*AMPK*) levels and decreased *NF-κB* and lectin-like oxidized LDL receptor 1 (*LOX1*) expression [96], suggesting influences on cellular stress responses and inflammatory pathways. Another randomized, double-blinded, placebo-controlled trial treated patients with idiopathic knee osteoarthritis with Krocina, a nanomicelle form of crocin (15 mg crocin/d for 4 mo) and conducted targeted qPCR of microRNAs in peripheral blood. Such crocin supplementation increased *miRNA-155* and decreased *miRNA-21* but did not affect *miRNA-146a* and *miRNA-223* [97]. These studies demonstrate the usefulness of qPCR applications for revealing the effect of carotenoids on inflammation at still physiologically achievable levels and other immune-related effects and allow meaningful comparisons between isolated carotenoids and carotenoids from food matrices.

#### Microarray analyses

A significant number of studies studying health-related aspects of carotenoids has used microarray techniques, mostly in the area of inflammation and cancer. In human nontumorigenic BEAS-2B lung bronchial epithelial cells, β-carotene (0.5, 1.5, 3 μM for 72 h) and β-apocarotenals (0.4 μM 4’-, 8’-, 10’-, 12’-apocarotenal for 72 h) were found to modulate a variety of genes as measured by transcriptomic microarray, largely involving retinoic acid (RA) signaling. RA-independent genes were also significantly affected by these compounds, such as those involved in apoptosis and TNF signaling [98]. β-Carotene (120 and 600 ppm in feed) supplemented to cigarette smoke-exposed A/J mice for 6 wk modulated genes in the lung tissue regarding inflammation pathways such as interleukin signaling and apoptosis. Notably, in this inflammation inhibition, β-carotene downregulated *Il-1α* and suspected downstream target growth-related oncogene 1 (*Gro-1*) [99]. In another study focusing on inflammation of the adipose tissue, lycopene isomers, (all-E)- and (5Z)-lycopene (2 μM for 24 h), influenced the transcriptome of 3T3-L1 preadipocytes in a study that revealed (all-E)- and (5Z)-lycopene significantly modulated (fold change >1.5, *P* < 0.05) 4821 and 3387 genes, respectively. Complementary qPCR shed light on the regulation of the inflammatory process, as these lycopene isomers downregulated proinflammatory *Il-6,* chemokine (*Mcp-1, chemokine (C-C motif) ligand 5* [*Ccl5*]), and acute phase protein (serum amyloid A 3 [*Saa3*]*, haptoglobin)* mRNA levels [48]. A treatment with apo-10’-lycopenoic acid, a potential lycopene metabolite (2 μM for 24 h) has also been found via microarray analysis to regulate the 3T3-L1 preadipocyte transcriptome by significantly modulating 607 genes (*P* < 0.05) notably related to ligand-dependent transcription of retinoid-target genes, in a manner similar to the method of action by all-trans RA. These changes induced by apo-10’-lycopenoic acid involved the reduction of proinflammatory cytokines (*Il-6, Il-1β*) in the adipocytes. Notably, in this study, apo-10’-lycopenoic acid appeared capable of transactivating RA receptors in the adipose tissue of RARE-luc mice [81]. These are important findings, as lycopene has largely been considered a non-provitamin A carotenoid, whereas these studies proposed effects that are in part vitamin A-like.

Fucoxanthinol, the deacetylated form of fucoxanthin, an algae-originating carotenoid, generated in the gastrointestinal tract (5 μM for 2 d), modulated the human pancreatic PANC-1 cancer cell transcriptome in pathways regarding the cell cycle, integrin, *AKT, MAPK, NRF2,* adipogenesis, transforming growth factor beta (*TGF-β*), signal transducer and activator of transcription *(STAT), or WNT* signals [100, 101]. Additionally, in mouse pancreatic KMPC44 cancer cells, fucoxanthinol (5 μM for 1 d) regulated inflammation and growth pathways, including *Mapk,* *Nf-κb,* protein kinase C (*Pkc*)*,* *Stat, Tgf-β,* and epidermal growth factor receptor (*Egfr*) [101]. Also, the apo-carotenoid crocin (10 μg/mL for 24 h) was found to modulate 723 genes (*P <* 0.05) involving cell-cycle signaling, reduction of cell viability, and induction of apoptosis in human pancreatic BXPC-3 cancer cells via microarray analysis [102].

Another area of investigation is the effects of carotenoids on adipose tissue. β-Carotene (150 mg/kg diet/d for 14 wk) could reduce adiposity and leptinemia in C57BL/6 mice through decreased expression of PPARγ-related genes (*Rxrα,* lipoprotein lipase [*Lpl*]*)*, as seen by microarray analysis of inguinal white adipose tissue. However, this reduction of adiposity and PPARγ activity depended on the presence of BCO1 and associated production of retinoids, as these results were only seen in wild-type mice but not reflected in BCO1 knockout mice [49]. Male Tsumura Obese, Diabetes (TSOD) mice were administered β-cryptoxanthin from enzyme-processed Satsuma mandarins (EPSM) at a dosage of 400 mg EPSM/kg bw/d (containing 0.8 mg β-cryptoxanthin/kg bw) for 8 wk to investigate the modulation of visceral fat. Epididymal adipose tissue, liver, and femoral muscle tissue of the TSOD were subjected to DNA microarray analysis to assess modulation by β-cryptoxanthin. Hepatic genes were involved in steroid metabolism, protein kinase activity, amino acid phosphorylation, and DNA replication initiation; adipocyte genes were related to the regulation of apoptosis, chemotaxis, immune system development; and finally, muscular genes corresponded to muscle contraction, lipid transport/fatty acid biosynthesis, hormone regulation, and wound healing [103].

Some studies utilized targeted RT-PCR microarrays with carotenoid treatment by focusing on gene expression related to nuclear receptors and cancer. For example, lycopene beadlets or tomato powder (10% w/w in diet for either treatment over 3 wk) were administered to male wild-type and BCO2^−/−^ mice in order to investigate hepatic nuclear receptor/coreceptor and stress/metabolism genetic modulation. Regarding nuclear receptors, both lycopene and tomato powder downregulated estrogen related receptor alpha (*Esrra*), histone deacetylase 3 (*Hdac3*), nuclear receptor coactivator 4 (*Ncoa4*), nuclear receptor subfamily 1 group D member 2 (*Nr1d2*), *Nr3c1*, *Ppparα*, and *Ppargc1β* as well as *Pparγ* in the case of lycopene in BCO2^−/−^ mice compared to wild-type. For stress/metabolism, lycopene and tomato powder suppressed cytochrome P450 family 1 subfamily B member 1 (*Cyp1b1*), cytochrome P450 oxidoreductase (*Por*), and uracil DNA glycosylase (*Udg*) in addition to crystallin alpha B (*Cryαb*), heat shock protein family A (*HspA5*), and cyclin-dependent kinase inhibitor 1A (*Cdkn1a*) by lycopene [104]. Another study of the same research group utilized RT-PCR array targeting genes involved in prostate carcinogenesis and cholesterol/lipoprotein metabolism in BCO2^−/−^ and transgenic adenocarcinoma of the mouse prostate (TRAMP) mice supplemented with lycopene beadlets or tomato powder (10% w/w in the diet for either treatment over 5 wk). For prostate carcinogenesis, lycopene decreased NK3 homeobox 1 (*Nkx3-1*) expression. Both lycopene and tomato powder increased several lipid metabolism-related genes (*Fasn, Acacα, Srebf1*, 3-hydroxy-3-methylglutaryl-CoA reductase [*Hmgcr*], and prostaglandin-endoperoxide synthase 1 [*Ptgs1*]) [105].

A randomized, double-blinded, placebo-controlled clinical trial known as the Molecular Effects of Nutrition Supplements (MENS) study utilized microarray technology on prostate tissue biopsies of men with low-risk prostate cancer supplemented with either lycopene (2x 15-mg tablets/d) or fish oil for 3 mo. While there were no significant differentially expressed genes between the lycopene and fish oil groups, there were some changes between the lycopene and placebo groups regarding *NRF2* oxidative stress [106]. These results demonstrate the capability of microarray analyses to reveal relations between carotenoids and their metabolites to health-relevant cellular signaling pathways in a more holistic manner compared to qPCR.

#### RNA-seq

A number of studies have incorporated RNA-seq to study the effect of various carotenoids on molecular pathways. Lycopene (200 mg/kg bw/d for 20 d) improved placental health of Sprague-Dawley rats receiving a high-fat diet (47% fat energy) by reducing placental oxidative stress and inflammation as well as increasing fetal growth. RNA-seq data showed that placental inflammation was reduced via lycopene-induced inhibition of the IL-17 pathway (*Il-17*, *Il-6*, *Tnf-α)*. Lycopene also reduced placental oxidative stress by promoting total antioxidant capacity as well as glutathione peroxidase (*GPx*) and glutaredoxin (*Grx*) activity. Furthermore, lycopene improved fetal development by increasing average fetal and litter body weight compared to the high-fat diet group [107]. In another animal study, lycopene administration (40 mg/kg diet/d for 35 d) influenced fat metabolism related genes in the liver, jejunum, and duodenum tissues of Xinghua breeding hens. RNA-seq performed on the liver transcriptome and lycopene significantly differentially modulated 158 genes, 89 downregulated and 69 upregulated genes, compared to the control group (*P <* 0.05). According to KEGG pathway analysis, these 158 genes were all involved in the biosynthesis of unsaturated fatty acids, retinol metabolism, drug metabolism, and metabolism of xenobiotics by cytochrome P450. Notably, RNA-seq indicated that lycopene increased hepatic *RARα*, *RXRα*, *PGC-1α*, and *PPARα* as well as decreased hepatic fatty acid-binding proteins (*FABP1* and *FABP10*). Complementary qPCR also found that lycopene increased duodenal *RARα* and jejunal *PPARγ, RXRα,* and *RXRγ* [108], emphasizing the manyfold interactions of lycopene with nuclear receptors.

As shown by RNA-seq data, astaxanthin, a marine-derived xanthophyll carotenoid present in salmon, ameliorated gut and liver statuses that were under challenge. Administration of supraphysiological levels of astaxanthin (5 μM for 7 h) inhibited *Helicobacter pylori*-induced activation of the wingless integration site (Wnt)/β-catenin pathway in human gastric epithelial AGS cells [109]. Astaxanthin (50 mg/kg bw/d for 10 wk) protected C57BL/6J mice from ethanol-induced liver injury in a model for alcoholic liver disease (ALD) by inhibiting the activity of the NOD-like receptor, toll-like receptor (TLR), and chemokine (MCP-1, MIP-2*)* signaling pathways [110]. RNA-seq data has shown that fucoxanthin (1 μM for 5 d) alleviated high-glucose-induced oxidative stress in SV40 MES 13 kidney mesangial cells by inhibiting the TLR, Hedgehog, and cAMP response element-binding protein (CREB) signaling pathways [111]. Fucoxanthin (1 μM and 5 μM for 3 d) has also been observed to promote Nrf2/antioxidant response element activity in lung Lech fibroblasts via RNA-seq [112], also emphasizing its involvement in oxidative stress related pathways.

As lutein and zeaxanthin are involved in the etiology of AMD and other eye diseases, studies have also evaluated their impact on gene expression. Zeaxanthin dipalmitate isolated from wolfberries (4 μM administered into vitreous humor for 9 d) alleviated retinal degeneration in C57BL/6J mice afflicted with retinis pigmentosa, an eye disease affecting the retina, by regulating several pathways as assessed by RNA-seq, including *Jak-Stat*, *Nf-κb*, *Tnf-α*, forkhead box O (*Foxo)*, and *Mapk* signaling cascade related genes [113]. Although there are limited human studies employing RNA-seq, the results reveal novel insights into the relation of carotenoids and a variety of pathways related to oxidative stress, inflammation, and further immune-related functions.

### Present gaps and perspectives

Transcriptomics has clearly been the omics technique the most applied to the research field of carotenoids and health-related outcomes. Though most studies have focused on targeted, i.e., qPCR employment, several studies have used microarray and RNA-seq. This includes *in vitro*, animal, and human studies, not only of frequently consumed carotenoids, such as α-carotene, β-carotene, β-cryptoxanthin, lycopene, lutein, and zeaxanthin but namely also of apo-carotenoids and some less frequently studied carotenoids. However, the potential of microarray and RNA-seq remains unrealized in human studies, though the animal studies mentioned above indicate the promise of these techniques. In humans, studies have been limited to the focus on prostate tissue and blood cell analysis, focusing on CVD, prostate cancer, and osteoarthritis. Though blood cells will possibly remain the target of choice, other cells such as buccal cells may also be used, though yielding a far lower number of cells. Another area of growth is also the use of miRNA and lncRNAs, though at least the former has been studied in connection with crocin in humans (*miRNA-21* and *miRNA-155* in relation to osteoarthritis, Table 1 [97]). Also, the use of further time-series transcriptomics related to the effect of carotenoids awaits further studies, which may increase comparability of findings to proteomics. This could be especially relevant for single dosing approaches with carotenoids due to the rapid absorption and turnover in certain plasma fractions of newly absorbed carotenoids in the plasma triacylglycerol-rich lipoprotein (TRL) fraction bloodstream of several hours [114,115] though their plasma half-life is considerably longer, up to several weeks [116]. However, time-dependent changes may trigger differential transcription factor or nuclear receptor activation. Clearly, the further advancement of technologies involving microarray and RNA-seq and their increasing affordability will aid in their future use in carotenoid related health research.

**TABLE 1:** Recent studies using transcriptomic techniques highlighting the beneficial role of carotenoids and their potential impact on relevant health outcomes

Study [reference] and main findings	Model	Technique	Design (approach)	Carotenoid	Dosage (duration)	Tissue	Disease state
***In vitro* studies**							
(all-E)- and (5Z)-Lycopene display similar biological effects on adipocytes [48]	3T3-L1 preadipocytes + (all-E)- or (5Z)-lycopene dissolved in THF/BHT. Control: vehicle (THF/BHT) only	1) Microarray 2) Transactivation assay 3) qPCR	N/A (Targeted and untargeted)	(all-E)- or (5Z)-Lycopene	2 μM (24 h)	Adipose tissue	Inflammation, obesity-related disorder

**Findings**	*1*)(all-E)- & (5Z)-Lycopene modulated the transcriptome of 3T3-L1 adipocytes: 4821 genes were regulated by (all-E)-isomer (2392 ↑, 1852 ↓). 3387 genes were regulated by (5Z)-isomer (1852 ↑, 1535 ↓) *2*)(all-E)- and (5Z)-Lycopene induced transactivation of PPARγ 3)(all-E)- and (5Z)-Lycopene modulated inflammatory process: ↓ phosphorylation levels of p65. ↓ TNFα-induced proinflammatory cytokine mRNA expression *4*)(all-E)- and (5Z)-Lycopene affected glucose uptake and insulin sensitivity in adipocytes: - ↓ 2-deoxyglucose uptake and AKT phosphorylation in 3T3-L1 adipocytes						

Lycopene inhibits proinflammatory cytokine and chemokine expression in adipose tissue [91]	1) *Ex vivo* cultures of mouse adipose tissue explants + lycopene subjected to TNF-α (15 ng/ mL) for 3 h. 2) Human preadipocytes 3T3-L1 + lycopene followed by a 3-h incubation with TNF-α (15 ng/mL)	qPCR	N/A (Targeted)	Lycopene	2 μM (24 h)	White adipose tissue	Inflammation, obesity

**Findings**	In mouse adipose tissue explants, lycopene ↓ expression of *Il-6* by 58%, ↓ expression of *Mcp*-1 by 20% In human preadipocytes 3T3-L1, lycopene ↓ expression of *Il-6* by 40%, ↓ expression of *Mcp-1* by 30%						

Lycopene attenuates LPS-induced TNF-α secretion in macrophages and inflammatory markers in adipocytes exposed to macrophage-conditioned media [92]	RAW 264.7 macrophages and 3T3-L1 preadipocytes + lycopene in tetrahydrofuran (THF) at 0.01%	qPCR	N/A (Targeted)	(All-E)-Lycopene	0.5 to 2 μM (time of cellular exposure-24 h)	Adipose tissue	Inflammation

**Findings**	Lycopene: ↓TNF-α in lipopolysaccharide-stimulated RAW 264.7 macrophages, ↓ secretion of TNF-α in RAW 264.7 macrophages, ↓ JNK phosphorylation in RAW 264.7 macrophages, ↑ adiponectin mRNA levels, ↓ inflammatory gene mRNA expression (*Il-6*, *Mcp-1*, *Il-1*, *Rantes*, *Cxcl1*, *CXCL10*, *SAA3*, and haptoglobin) and remodeling gene mRNA expression (*Mmp3* and *Mmp9*) in 3T3-L1 adipocytes						

Lycopene supplementation to serum-free maturation medium improves *in vitro* bovine embryo development and quality and modulates embryonic transcriptomic profile [241]	Bovine embryo- oocyte *in vitro* maturation (IVM)	RNA-seq	N/A (Untargeted)	Lycopene	0.2 μM (8 d)	Oocytes (bovine embryo)	Oxidative stress

**Findings**	Lycopene treatment during IVM: ↓ expression of *CASP3* and *BAX*, ↑ expression of *BCL2*, ↓ expression of *IκBKB* gene						

Synergistic protection of quercetin and lycopene against oxidative stress via SIRT1-Nox4-ROS axis in HUVEC cells [242]	Human umbilical vein endothelial cells (HUVECs)	RNA-seq and RT-PCR	N/A (Targeted and untargeted)	Quercetin-lycopene (5:1)	5 μM (12 h)	Human umbilical vein endo-thelial cells	Endothelial dysfunction

**Findings**	Quercetin-lycopene combination: ↓ *IL-17*, ↓ *NF-κB*						

β-Carotene and apocarotenals promote retinoid signaling in BEAS-2B human bronchioepithelial cells [98]	BEAS-2B human bronchial epithelial cells	qRT-PCR	N/A (Targeted)	β-Carotene apocarotenals (4'-, 8'-, 10' -, 12'-)	0.5, 1.5, 3 μM (72 h) 0.4 μM (72 h)	Model of human lung	Cancer

**Findings**	β-Carotene affected RA signaling: ↑ *RARβ*, *RARRES 1*, *MEOX1*, *FOXA1*, *GATA6*, *HOXC10*, *HOXB6*, *HOXA7*, *HLXB9*, *DLX4*, *DLX2*, *LHX3*, and *SIX6*. ↓ *IRX4*. Apocarotenal: ↑ *RARRES 1*, *GATA6*, *HOXB6* and *RARβ*. ↓ *CRABP2* and *RARRES 3* Both β-carotene & apocarotenals affected apoptosis signaling: ↓ TNF signaling						

Epigenomic, trans-criptomic, and protective effect of carotenoid fucoxanthin in high glucose-induced oxidative stress in Mes13 kidney mesangial cells [111]	Mes13 kidney mesangial cells	RNA-seq	N/A (Untargeted)	Fucoxanthin	1 μM (5 d)	Kidney	Oxidative stress, diabetic nephropathy

**Findings**	Fucoxanthin-attenuated high glucose-induced oxidative stress: ↓ TLR cascades- TLR 4,7,8,9, ↓ Hedgehog signaling, ↓ PKA-mediated CREB phosphorylation						

Protective effects of carotenoid fucoxanthin in fibroblasts cellular senescence [112]	LECh4(81) fibroblasts	RNA-seq	N/A (Untargeted)	Fucoxanthin	1 μM & 5 μM (3 d)	Model of human lung	Oxidative stress

**Findings**	Fucoxanthin: ↑ Nrf2/ARE antioxidant activity, ↓ ROS, ↓ UPC2, ↓ mitochondrial oxidative phosphorylation, ↑ ABC transporters						

A marine carotenoid of fucoxanthinol accelerates the growth of human pancreatic cancer PANC-1 cells [100]	Human pancreatic cancer PANC-1 cells; human colorectal cancer DLD-1 cells	Microarray, western blot, qPCR	N/A (Targeted and untargeted)	Fucoxanthinol	5 μM (2 d)	Model of human pancreas	Pancreatic cancer

**Findings**	Fucoxanthinol (microarray): pathways of cell cycle, integrin, *AKT*, *MAPK*, *NRF2*, adipogenesis, *TGF-β*, *STAT*, or Wnt signals Fucoxanthinol (western blot): ↑ expression of integrin β1 and PPARγ as well as the activation of pFAK(Tyr397), pPaxillin(Tyr31), and pAKT(Ser473) Fucoxanthinol (qPCR): ↑ expression of *FYN*, a downstream target of integrin subunits						

A fucoxanthinol induces apoptosis in a pancreatic intraepithelial neoplasia cell model [101]	Mouse pancreatic cancer KMPC44 cells	Microarray, western blot	N/A (Targeted and untargeted)	Fucoxanthinol	5 μM (1 d)	Model of pancreatic cancer	Pancreatic cancer

**Findings**	78 upregulated and 7 downregulated genes: growth and inflammation (*EGFR*, *GPCR*, integrin, Jun, *Mapk*, *Nf-κb*, *PI3k/Akt*, *Pkc*, *Ras*, *Stat*, *Tgf-β*, and *Wnt*)						

Inhibitory effect of astaxanthin on gene expression changes in helicobacter pylori-infected human gastric epithelial cells [109]	Human gastric epithelial AGS cells	RNA-seq, RT-PCR	N/A (Targeted and untargeted)	Astaxanthin	5 μM (7 h)	Model of gastric epithelial cell	Gastric cancer; *H. pylori* infection

**Findings**	Astaxanthin: Reversed *H. pylori*-induced activation of Wnt/β-catenin pathway, ↓ *PORCN*, *FOSL1*, *MYC*, *SMOX*, ↑ *BAMBI,SMAD4*						

Dietary crocin is protective in pancreatic cancer while reducing radiation-induced hepatic oxidative damage [243]	Bxpc-3 and Capan-2 human pancreatic cancer cell lines	Microarray, western blot	N/A (Targeted and untargeted)	Crocin	10, 20, 30 and 40 μg/mL (24 h)	Model of human pancreatic cancer cell lines	Pancreatic cancer, oxidative stress

**Findings**	Crocin: Reduced cell viability of BXPC3 and Capan-2 by triggering caspase signaling via the downregulation of Bcl-2 Modulated the expression of cell-cycle signaling proteins *P53*, *P21*, *P27*, *CDK2*, *c-MYC*, *CYT-c*, and *P38.* Induced apoptosis by inducing the release of cytochrome c from mitochondria to cytosol of BXPC3 and Capan-2 cells Showed protection against radiation-induced hepatic oxidative damage in mice bearing pancreatic tumors, by reducing the levels of hepatic toxicity and preserving liver morphology						

***In vivo* animal studies**							
Lycopene and tomato powder supplementation similarly inhibit high-fat diet induced obesity, inflammatory response, and associated metabolic disorders [93]	Male C57BL/6J mice (*n*=40)	qPCR	4 groups (*n*=10/group) 1st: Control diet 2nd: high fat diet (HFD 45% fat) 3rd: HFD + lycopene 4th: HFD + tomato powder (Targeted)	Lycopene and tomato powder	Lycopene 10 mg/kg Tomato powder 10 mg/kg diet/d (12 wk)	Liver, adipose tissue	Inflammation, obesity

**Findings**	Lycopene and tomato powder supplementation: ↓ HFD-induced proinflammatory cytokine mRNA expression in the liver and in the epididymal adipose tissue (*Tnf-α*, *Mcp-1*, *Il-6*, *Ccl2*, and *Ccl5*), ↓ Hepatic gene involved in lipid metabolism (*Acacα*, *Fasn*, *Srebp-1c)*, ↓ mRNA level of *Pparγ*, *Cd36*, *aP2* and *Lpl*, ↓ phosphorylation levels of IκB, and p65						

Lycopene inhibits proinflammatory cytokine/ chemokine expression in adipose tissue [91]	Male C57BL/6J mice (*n*=6)	qPCR	2 groups (*n*=6/group): -HFD (35% fat) -normal chow supplemented with lycopene (Targeted)	Lycopene	2 μM; conc. in human plasma by consumption of tomato sauce (6 wk)	White adipose tissue	Obesity

**Findings**	Compared to HFD, lycopene ↓ expression of mRNA of the different markers, by 50% for *Il-6*, −6% for *Mcp-1*, 50%, for *Il-1β*						

Effect of lycopene on oral squamous cell carcinoma cell growth by inhibiting IGF1 pathway [244]	Athymic nude mice (*n*=10)	Microarray, Western blot, qRT-PCR	2 groups (*n*=5/group) Control: CAL-27 cells injected subcutaneously. LYC: CAL-27 + 16 mg LYC/kg bw (Targeted and untargeted)	Lycopene	16 mg/kg bw twice per week (22 d)	Oral tumor	Oral squamous cell carcinoma (OSCC)

**Findings**	Lycopene: 997 and 572 genes were up- and downregulated in OSCC tissues, respectively ↓ Igf1 signaling pathway: *Igf1*, *Igfbp1*, *Jun*, and *Foxo1*						

β-Carotene-9′,10′-oxygenase status modulates impact of dietary tomato and lycopene on hepatic nuclear receptor–, stress-, and metabolism-related gene expression [104]	Male wild-type and BCO2^−/−^ mice (C57BL/6 background) (*n*=66)	RT-PCR array	6 groups (*n*=11/group) wild-type (WT) BCO2^−/−^ WT + tycopene BCO2^−/−^ + tycopene WT + tomato BCO2^−/−^ + tomato (Targeted)	Lycopene	0.25% w/w redivio (10% lycopene) or 10% w/w tomato powder in diet; equivalent to 462 and 384 mg lycopene/kg of diet, resp. (3 wk)	Liver	nonalcoholic steatohepatitis/hepatocarcinogenesis

**Findings**	Targeted for nuclear receptors/coreceptors: ∗Tomato in Bco2^−/−^ mice/compared to WT: ↓ expression of *Esrra*, *Hdac3*, *Ncoa4*, *Nr1d2*, *Nr3c1*, *Ppar-α*, and *Ppargc1β* ∗Lycopene in Bco2^−/−^ mice/compared to WT: ↓ expression of *Esrra*, *Hdac3*, *Ncoa4*, *Nr1d2*, *Nr3c1*, *Ppar-α*, *Ppargc1β*, and *Ppar-γ* Targeted for stress/metabolism: ∗Tomato in Bco2^−/−^ mice/compared to WT: ↓ expression of *Por* and *Ung*, ↑ expression of *Cyp1b1* and *Egr1* ∗Lycopene in Bco2^−/−^ mice/compared to WT:↓ expression of *Cryαb*, *HSPa5*, *CDKn1a*, *Cyp1b1*, *Por*, and *Ung*						

β-Carotene oxygenase 2 genotype modulates the impact of dietary lycopene on gene expression during early TRAMP prostate carcinogenesis [105]	TRAMP^/+^, BCO2^−/−^ mice (C57BL/6 background)	RT-PCR	Control diet: TRAMP^/+^, TRAMP^/-^ × BCO2^+/+^, BCO2^−/−^ Lycopene beadlets: TRAMP^/+^, TRAMP^/−^ x BCO2^+/+^, BCO2^−/−^ Tomato powder: TRAMP^/+^, TRAMP^/−^ x BCO2^+/+^, BCO2^−/−^ (Targeted)	Lycopene	0.25% w/w beadlet (10% lycopene) or 10% w/w tomato powder in diet; equivalent to 462 and 384 mg lycopene/kg of diet, resp. (5 wk)	Prostate	Prostate cancer

**Findings**	Lycopene: ↓ gene expression related to carcinogenesis (*Nkx3-1*) Tomato feeding: ↑ gene expression related to circadian regulation (*Arntl*) Tomato and/or lycopene: ↑ gene expression related to lipid metabolism (*Fasn,Acacα*, *Srebf1*, *Hmgcr*, and *Ptgs1*)						

Lycopene modulates placental health and fetal development under high-fat diet during pregnancy of rats [107]	Sprague-Dawley rats (*n*=48)	RNA-seq, qRT-PCR	3 groups (*n*=16/group) 1st: Control 2nd: HFD (47.13% fat) 3rd: HFD + lycopene (Targeted and untargeted)	Lycopene	200 mg/kg of diet (4.94 mg/d/rat) (20 d)	Placenta	pregnancy-related complications (placental oxidative stress and inflammation, fetal development)

**Findings**	Lycopene in HFT group: ↓ *Il-17*, *Il-6,* and *Tnf-α* in placenta through the IL-17 pathway, ↓ ROS, ↓ H_2_O_2_ levels, ↑ *Grx* gene and protein expression in the placenta, ↑ *GPx* and *T-aoc* levels, ↑ expression of Lep gene and protein, ↑ level of leptin Fetal development: ↑ average fetal weight and ↑ fetal litter weight						

Lycopene supplementation regulates the gene expression profile and fat metabolism of breeding hens [245]	Xinghua breeding hens	RNA-seq qPCR	4 groups (*n*=30/group; replicated 6 times): Control: broken rice and soybean meal 20 mg LYC/kg diet/d 40 mg LYC/kg diet/d (used for RNA-seq) 80 mg LYC/kg diet/d (Targeted & untargeted)	Lycopene	40 mg/kg diet (35 d)	Liver	Fat metabolism

**Findings**	RNA-seq: 158 DEGs- 69 upregulated genes and 89 downregulated genes, modulated biosynthesis of fatty acids and retinol metabolism Liver qPCR: ↑ *PGC1α*, *PPARα*, *RXRα*, *RARα* Jejunum qPCR: ↑ *PPARγ*, *RXRα*, *RXRγ*,↓ *FABP1*, *FABP10*						

Apo-10'-lycopenoic acid impacts adipose tissue biology via the retinoic acid receptors [81]	Adult male C57BL/6J mice (*n*=6, *ex vivo* experiments) RARE-luc mice (*in vivo* RAR transactivation experiments)	Microarray qPCR	Both of C57BL/6J and RARE-luc mice were divided into 2 groups (*n*=2–4 animals/group): 1st: standard chow diet 2nd: high fat diet (35% fat) RARE-luc mice (*in vivo* RAR transactivation experiments) (Targeted and untargeted)	Apo-10'-lycopenoic acid	4 h following injection of C57BL/6 male mice: C57BL/6 male mice (*n*=3) injected i.v. with 50 mg/kg bw lycopene (24 h of treatment of RARE-luc mice) Explants of adipose tissues from mice who consumed a HFD or normal chow diet treated with medium containing 2 μmol/L apo-10-lycopenic acid in THF/BHT at 0.1% in final conc. (24 h)	Adipose tissue	Biological activities via the retinoic acid receptors (RAR)

**Findings**	Apo-10'-lycopenoic acid modulated the transcriptome of 3T3-L1 adipocytes in a manner similar to ATRA (607 genes were regulated by apo-10-lycac (↑ 439 and ↓ 168) Apo-10'-lycopenoic acid ↓ proinflammatory markers in adipose tissue and adipocytes (IL-6 [−20%], IL-1β [−40%], IL-6 [−20%], and IL-1β [−60%]) Apo-10'-lycopenoic acid transactivated RAR *in vivo*, *in vitro* and modulates transcription of RAR target genes in adipose tissue						

Regulatory mechanisms of β-carotene and BCMO1 in adipose tissues: A gene enrichment-based bioinformatics analysis [246]	Female B6129SF mice	Microarray	24 mice were divided into 4 groups (*n*=6/group): *Bcmo1*^+/+^ with control diet. *Bcmo1*^+/+^ with β-carotene diet *Bcmo1*^−/−^ under control diet. *Bcmo1*^−/−^ with β-carotene diet (Untargeted)	β-Carotene	150 mg β-carotene /kg diet (14 wk)	Adipose tissue	Impact of molecules derived from β-carotene on the physiological functions of fat cells

**Findings**	β-carotene diet in *Bcmo1*^+/+^, as compared to *Bcmo1*^+/+^ with control diet: Upregulated 9 genes and downregulated 33 genes β-carotene diet in *Bcmo1*^−/−^, as compared to *Bcmo1*^−/−^ with control diet: Upregulated 78 genes and downregulated 89 genes						

β-Carotene reduces body adiposity of mice via BCMO1 [49]	Wild-type and *Bcmo1*^−/−^ mice (C57BL/6 background)	qPCR, western blot, microarray	4 groups (*n*=6 animals/group): Wild-type: pelletized diet containing 1500 IU vitamin A/kg and 10% energy as fat. Wild-type: pelletized diet containing 1500 IU vitamin A/kg, 10% energy as fat, and β-carotene. Bcmo1^−/−^ mice: pelletized diet containing 1500 IU vitamin A/kg and 10% energy as fat. Bcmo1^−/−^ mice: pelletized diet containing 1500 IU vitamin A/kg, 10% energy as fat, and β-carotene (Targeted and untargeted)	β-Carotene	150 mg/kg diet (14 wk)	Adipose tissue	Inflammation, obesity

**Findings**	β-Carotene diet in *Bcmo1*^+/+^, as compared to *Bcmo1*^+/+^ with control diet: ↓ PPARγ and PPARγ target genes in adipose tissue						

Transcriptomics does not show adverse effects of β-carotene in A/J mice exposed to smoke for 2 weeks [99]	A/J mice (*n*=360)	Microarray qPCR	6 groups (*n*=60/group): 1st: cigarette smoke + 0 ppm of β-carotene in diet. 2nd: cigarette smoke + 120 ppm of β-carotene in diet. 3rd: cigarette smoke + 600 ppm of β-carotene in diet. 4th: Control 0 ppm of β-carotene in diet. 5th: Control 120 ppm of β-carotene in diet. 6th : Control 600 ppm of β-carotene in diet (Targeted and untargeted)	β-Carotene	Treatment with β-carotene: 120 and 600 ppm β-carotene in feed (6-wk) Exposure to cigarette smoke: 140 mg total suspended particulates/m^3^ (last 2 wk)	Lung	Oxidative stress, inflammation

**Findings**	β-Carotene: ↓ inflammation via reducing expression of *Gro I* and *IL-1*, ↓ extracellular matrix (ECM) degradation, via reducing *Mmp12* and *Mmp3* expression						

Gut microbiota regulation and anti-inflammatory effect of β-carotene in dextran sulfate sodium-stimulated ulcerative colitis in rats [212]	Specific pathogen-free male SD rats	qPCR; 16S rRNA for microbiota	4 groups (*n* = 6/group) 1st : Normal diet (control group) 2nd: β-carotene supplementation 3rd : Dextran sulfate sodium (DSS), ulcerative colitis model 4th : Dextran sulfate sodium and β-carotene (Targeted and untargeted)	β-Carotene	50 mg/kg bw (1 wk)	Gut microbiota	Ulcerative colitis, inflammation

**Findings**	β-Carotene: Inhibited the expression of proinflammatory factors (induced by DSS), ↓ expression of *p65*, *p38*, *Erk*, and JNK, ↓ severity of colitis in rats (body weight, colon length, disease activity index)						

Ameliorative effect of spinach on nonalcoholic fatty liver disease induced in rats by a high-fat diet [95]	Sprague-Dawley Rats (*n*=44)	qRT-PCR	6 groups: 1st: NC (standard diet, n=6) 2nd: N2.5 (standard diet + 2.5% spinach, n=8) 3rd : N5 (standard diet + 5% spinach, n=8) 4th : HC (high-fat diet, n=6) 5th : H2.5 (high-fat diet + 2.5% spinach, n=8) 6th : H5 (high-fat diet + 5% spinach, n=8) (Targeted)	Spinach powder (1750 μg total carotenoids/g; 228 μg neoxanthin/g 292 μg violaxanthin/g, 944 μg lutein/g 46 μg α-carotene/g 225 μg β-carotene/g	5% spinach in diet = 53-56 μg of total carotenoids/d (5 wk) 2.5% spinach in diet = 20-24 μg of total carotenoids/d (5 wk)	Liver	NAFLD

**Findings**	Accumulation of lutein, α-carotene, and β-carotene in the liver: ↑ expression of genes involved in the metabolism of FAs and cholesterol, via overexpression of PPARs						

Comparative transcriptome analyses provide potential insights into the molecular mechanisms of astaxanthin (Ax) in the protection against alcoholic liver disease in mice [110]	C57BL/6J mice (*n*=48)	RNA-seq qRT-PCR	4 groups (*n*=12/group) 1st: Lieber–DeCarli liquid diet 2nd: Lieber–DeCarli liquid diet & Ax 3rd : ethanol-containing Lieber–DeCarli liquid diet 4th : ethanol-containing Lieber–DeCarli liquid diet and Ax (Targeted and untargeted)	Astaxanthin	50 mg/kg bw (10 wk)	Liver	Alcoholic liver disease

**Findings**	Astaxanthin: ↓ ALD-liver injury via inhibition of the NOD-like receptor, TLR, and chemokine signaling pathways NOD-like: ↓ *Nlrp3*, caspase-1, *Il-1α*, *Il-1β*, *Il-18* TLR: ↓ *Tlr2*, *Tlr4,Myd88*. Chemokine: ↓ *Mcp-1*, *Mp-2*						

Astaxanthin (Ax) attenuates hepatic damage and mitochondrial dysfunction in non-alcoholic fatty liver disease by up-regulating the FGF21/PGC-1α pathway [94]	C57BL/6J mice (*n*=42)	qRT-PCR	(*n*=7/group) Control: standard chow diet. HFD group: HFD, saline by gavage every 2 d in last 10 wk. HFD + Ax group: HFD; 10, 30, and 60 mg/kg of Ax by gavage every 2 d in last 10 wk. Negative group: HFD, tail-vein injection of control-siRNA 8× during first 4 wk. siRNA-FGF21 group: HFD, tail-vein injection of FGF21-siRNA 8× during first 4 wk. siRNA-FGF21 + Ax group: HFD, tail-vein injection of FGF21-siRNA 8× during first 4 wk, received 60 mg/kg of Ax by gavage every 2 d during last 10 wk. (Targeted)	Astaxanthin	10, 30, 60 mg/kg by gavage per 2 d (12 wk)	Liver	NAFLD

**Findings**	Astaxanthin protected the liver of NAFLD mice by regulating altered lipid metabolism, fibrosis, and inflammation: ↓ expression of *Bax* and caspase 9, ↓ levels of TNF-ɑ, IL-1β, and iNOS, ↓ level of hepatic collagen I, TGF-β1, ɑ-SMA, and CTGF, ↑ expression of *Nrf1* and *Tfam*, ↑ expression of *Fgf21* and *Pgc-1α*						

Astaxanthin-shifted gut microbiota is associated with inflammation and metabolic homeostasis in mice [210]	C57BL/6J mice (*n*=80)	qPCR; 16S rRNA for microbiota	C57BL/6J mice per sex (female and male) grouped into 4 treatments (8 groups in total considering both sexes (*n*=10 mice/group) KO-CONT (*Bco2*^−/−^ mice fed control) ×2 KO-ASTX (*Bco2*^−/−^ mice fed astaxanthin) ×2 WT-CONT (WT fed control) ×2 WT-ASTX (WT fed astaxanthin) ×2 (Targeted and untargeted [16S])	Astaxanthin	0.04% wt/wt in diet (8 wk)	Gut microbiota	Inflammation, oxidative stress, and metabolic homeostasis

**Findings**	*Bco2*^−/−^ mice fed astaxanthin had 10-fold more astaxanthin than WT mice fed astaxanthin in liver, resulting in: ↑ 27% of plasma glucagon-like peptide 1 in male KO mice than the WT mice ↓ 53% of plasma glucagon in male KO mice than the WT mice ↓ 30% of IL-1β in male KO mice than the WT mice ↓ 23% of colon NOD-, LRR- and pyrin domain-containing protein 3 (NLRP3) inflammasome activation in male KO mice vs. WT mice						

Astaxanthin prevents alcoholic fatty liver disease by modulating mouse gut microbiota [209]	C57BL/6J mice (*n*=60)	qPCR; 16S rRNA for microbiota	Five groups (*n*=12 mice/group): -1st: normal standard growth diet for 16 wk. -2nd: high-fat liquid diets (HFT, 35% fat, 18% protein, 47% carbohydrates) for 16 wk. -3rd: HFT for 2 wk acclimation, then HFT combined with the astaxanthin (AST group, 50 mg/kg bw) for 12 wk. -4th: HFT 2 wk acclimation, then HFT with ethanol-containing (Et group, 5% ethanol v/v, for 36% of the total caloric intake) for 12 wk. -5th: HFT for 2 wk acclimation, then HFT with ethanol plus astaxanthin (EtAST group) for 12 wk (Targeted and untargeted [16S])	Astaxanthin	50 mg/kg bw (12 wk)	Liver	alcoholic fatty liver disease

**Findings**	Astaxanthin: ↓ inflammation (*Il-1α*, *Mip-2*, *Il-6*, and *Tnf-α)*,↓ excessive lipid accumulation (TG, LDL, HDL, and TC), ↓ serum markers of liver injury (ALT and AST).						

Wolfberry-derived zeaxanthin dipalmitate delays retinal degeneration in a mouse model of retinitis pigmentosa through modulating STAT3, CCL2 and MAPK pathways [113]	C57BL/6J mice (*n*=82)	RNA-seq and western blot	Three groups: 1st (*n*=26): treated with vehicle 2nd (*n*=29): treated with zeaxanthin dipalmitate 3rd (*n*=27): WT mice (Targeted and untargeted)	Zeaxanthin dipalmitate	Injection of 4 μM zeaxanthin dipalmitate into vitreous humor (9 d)	Eye	Retinitis pigmentosa

**Findings**	Zeaxanthin dipalmitate modulated the Jak-STAT, NF-κB, TNFα, FoxO & MAPK signaling pathways: JAK-STAT: ↓ *Lif, Stat3, Socs3, Cish, Gfap* NF-κB: ↓ *Nik, Nf-κb, Gaddd45β, Mip-1β* TNF: ↓ *Nik, Nf-κbp65, Ccl2, Cxl10, Lif, Socs3, Fos, Bcl2* FoxO: ↑ *Igf1, PI3k; ↓Bcl-6,Gadd45* MAPK: ↓ *p-Erk/Erk, p-P38/P38* ratios						

Mechanism of visceral fat reduction in tsumura suzuki obese, diabetes (TSOD) mice orally administered β-cryptoxanthin (CRX) from Satsuma mandarin oranges [103]	Male Tsumura Suzuki non-obese, diabetes (TSNO) mice (*n*=12); Tsumura obese, diabetes (TSOD) mice (*n*=12)	Microarray qRT-PCR	Tsumura Suzuki non-obese and Tsumura Suzuki obese split into 4 groups (*n*=6/group): TSNO/-CRX TSNO/+CRX TSOD/-CRX TSOD/+CRX (Targeted and untargeted)	β-Cryptoxanthin from enzyme-processed Satsuma mandarin (EPSM)	400 mg EPSM/kg bw (0.8 mg β-cryptoxanthin/kg BW) (8 wk)	Adipose tissue, liver, muscle	Obesity

**Findings**	β-Cryptoxanthin in TSOD mice: ↑ expression of *Hmgcs1*, *Cyp51*, *Idi1*, ↓ expression of *Hdlbp* and *Abca1*						

***In Vivo* Human Studies**							
Effects of crocin and saffron aqueous extract on gene expression of SIRT1, AMPK, LOX1, NF-κB, MCP-1 in patients with coronary artery disease: RCT [96]	Coronary artery disease (CAD) patients aged 40–65 (*n*=65)	qPCR	Group 1: Crocin (*n*= 22) Group 2: Saffron aqueous extract (SAE) (*n*=23) Group 3: Placebo (*n*=20) (Targeted)	Crocin	30 mg crocin/d or 30 mg SAE/d (8 wk)	Peripheral blood mononuclear cells	CAD

**Findings**	Crocin: ↑ *SIRT1*, *AMPK*; ↓ *LOX1*, *NF-κB*						

Significant effect of crocin on the gene expression of microRNA-21 and microRNA-155 in patients with osteoarthritis [97]	Patients (ages 40–75 y) with idiopathic knee osteoarthritis (*n*=35)	qPCR (microRNAs)	Krocina, nanomicelle form of crocin (*n*=18) Placebo (*n*=17) (Targeted)	Crocin	15 mg crocin/d (4 mo)	Peripheral blood of OA patients	Osteoarthritis (OA)

**Findings**	Crocin: ↑ microRNA-155, ↓ microRNA-21, No significant changes in microRNA-146a and microRNA-223						

Gene expression and biological pathways in tissue of men with prostate cancer in a randomized clinical trial of lycopene and fish oil supplementation [106]	Males with low-risk prostate cancer (*n*=84)	Microarray	MENS study: Lycopene (*n*=29); fish oil (1098 mg EPA and 549 mg DHA FAs (*n*=27) Placebo (*n*=28) (Untargeted)	Lycopene	Two 15 mg lycopene (Lyc-O-Mato)/d (3 mo)	Prostate tissue biopsy	Prostate cancer

**Findings**	Lycopene vs. fish oil: No significant changes Lycopene vs. placebo (*P* value not corrected for multiple comparisons): Modulation of Nrf2-mediated oxidative stress response						

Abbreviations: ABC, ATP-binding cassette; Abca1, ATP-binding cassette subfamily A member 1; Acacα, acetyl-CoA carboxylase alpha; Akt, protein kinase B; ALT, alanine aminotransferase; AMPK, 5’-adenosine monophosphate-activated protein kinase; aP2, adipocyte fatty acid-binding protein; ARE, antioxidant responsive element; Arntl, Aryl hydrocarbon receptor nuclear translocator like; α-SMA, spinal muscular atrophy; AST, aspartate aminotransferase; ATRA, all-trans retinoic acid; BAMBI, BMP and activin membrane-bound inhibitor homolog; Bax, Bcl-2-associated X protein; Bcl-2, B-cell lymphoma 2; BCO, β-carotene oxygenase; BHT, butylated hydroxytoluene; BMP, bone morphogenetic proteins; CASP3, cysteine-aspartic acid protease 3; CCL, chemokine (C-C motif) ligand; Cd36, cluster of differentiation 36; CDK2, cyclin-dependent kinase 2; CDKn1a, cyclin-dependent kinase inhibitor 1a; CISH, cytokine-inducible SH2-containing protein; CRABP2, Cellular retinoic acid-binding protein 2; CREB, cAMP response element binding protein; Cryαb, crystalline-α B; CXCL, chemokine (C-X-C motif) ligand; Cyp1b1, cytochrome P450 1b1; Cyp51, lanosterol 14-α-demethylase; DEG, differentially expressed gene; DLX4, distal-less homeobox 4; EGFR epidermal growth factor receptor; Egr1, early growth response 1; ERK, extracellular signal-regulated kinase; Esrra, estrogen-related receptor-α; FA, fatty acid; FABP, fatty acid-binding protein; Fasn, fatty acid synthase; FGF21, fibroblast growth factor 21; FOSL1, FOS like 1; Fox, forkhead box; FYN, proto-oncogene tyrosine-protein kinase; GADD45β, growth arrest and DNA damage-inducible 45 beta; GATA6, GATA binding protein 6; GFAP, glial fibrillary acidic protein; Grx, glutaredoxin; GPCR, G-protein-coupled receptor; Gro I, growth-regulated oncogene I; GPx, glutathione peroxidase; Hdac3, histone deacetylase 3; Hdlbp, high-density lipoprotein binding protein; HLBXB9, homeobox HB9; HMGCs1, 3-hydroxy-3-methylglutaryl-CoA synthase 1; HOX, homeobox; HSPa5, heat shock protein 5; Idi1, isopentenyl-diphosphate δ isomerase 1; IGF1, insulin-like growth factor 1; IGFBP1, IGF binding protein-1; IL, interleukin; iNOS, inducible nitric oxide synthase; IRX4, Iroquois homeobox 4; IκB, IkappaB kinase; JAK, Janus-activated kinase; JNK, c-Jun N-terminal kinase; JUN, transcription factor Jun/AP-1; KO, knockout;LHX3, LIM homeobox 3; LIF, leukemia inhibitory factor; LOX1, lectin-like oxidized LDL receptor 1; Lpl, lipoprotein lipase; MAPK, mitogen-activated protein kinases; MCP-1, monocyte chemotactic protein 1; MEOX1, mesenchyme homeobox 1; MIP-1β, macrophage inflammatory protein 1 beta; MMP, matrix metallopeptidase; MYC, myelocytomatosis oncogene; MyD88, myeloid differentiation primary response 88; NAFLD, nonalcoholic fatty liver disease; Ncoa4, nuclear receptor coactivator 4; NF-κB, nuclear factor-kappa B; NK, natural killer; Nkx3-1, NK3 homeobox 1; NLRP3, NOD-like receptor family, pyrin domain containing 3; NOD, nucleotide-binding oligomerization domain; Nr1d2, nuclear receptor subfamily 1 group D member 2; Nr3c1, glucocorticoid receptor; Nrf2, nuclear factor (erythroid-derived 2)-like 2; pFAK, phosphorylated focal adhesion kinase; PGC-1α, peroxisome proliferator-activated receptor gamma coactivator 1-alpha; PI3K, phosphatidylinositol 3-kinase; PKA, protein kinase A; PKC, protein kinase C; Por, P450 (cytochrome) oxidoreductase; PORCN, porcupine O-acyltransferase; Ppar, Peroxisome proliferator-activated receptor; Ppargc1b, peroxisome proliferator-activated receptor-gamma coactivator 1 β; Ptgs1, prostaglandin-endoperoxide synthase 1; RANTES, Regulated upon Activation, Normal T Cell Expressed and Secreted; RARRES, retinoid acid (RA) receptor responder 1; RARα, retinoic acid receptor alpha; Ras, rat sarcoma virus; RCT, randomized clinical trial; ROS, reactive oxygen species; RXRα, retinoid X receptor alpha; SAA3, serum amyloid A 3; SIRT1, Sirtuin 1; SMAD4, mothers against decapentaplegic homolog 4; SMOX, spermine oxidase; SOCS3, suppressor of cytokine signaling 3; SREBP-1c, sterol regulatory element-binding transcription factor 1; STAT, signal transducer and activator of transcription; T-AOC, total antioxidant capacity; TC, total cholesterol; TFAM, mitochondrial transcription factor A; TG, triglyceride; TGF-β, transforming growth factor beta; THF, tetrahydrofuran; TLR, toll-like receptor; TNFα, tumor necrosis factor alpha; TRAMP, transgenic adenocarcinoma of the mouse prostate; Udg, uracil DNA glycosylase; UPC2, sterol uptake control protein 2; Wnt, wingless integration site; WT, wild type.

## Effects of Carotenoids as Assessed by Proteomics

### Methodological overview

#### 2D-DIGE.

2D-DIGE is an extension of 2D-PAGE (polyacrylamide gel electrophoresis), allowing the detection of multiple protein extracts on one gel and comparison between gels by the introduction of a common internal standard. It is historically the older method of proteomics when compared to gel-free alternatives such as nano-LC, with 2D-DIGE being introduced in 1975 by O’Farrell and Klose [117,118]. The protein of interest from a tissue or cell first need to be isolated, then first separated according to its charge, i.e., its isoelectric point (isoelectric focusing, first dimension), then according to their mass (second dimension), typically by SDS-PAGE due to the adjustable pore sizes relying on concentrations of acrylamide and a cross-linker. The advantage of 2D-DIGE over other approaches consists of a general high resolution (separation of a large number of protein spots), good sensitivity, linearity of the fluorescence dyes employed, and accurate quantification of the proteins, as well as low intergel variability due to the use of internal standards [119]. For detailed information regarding the isolation and purification of proteins, the reader is referred to other articles [11,57]. In brief, around 50 μg protein may be required. Major proteins, such as those abundant in plasma but of no perceived relevance (such as albumin and immunoglobulins), can be removed by commercial depletion columns [11].

Another potential option for proteomics is to analyze cellular subfractions, such as cytosolic proteins and membrane proteins [53], though even these may be further separated to identify proteins in the nucleus. This may improve identification of proteins and their functionality in respective cellular locations. This may be of interest for carotenoids considering their association with lipophilic membranes that may produce more pronounced changes in certain subcellular fractions, and this has been applied already to study effects in plant tissues [58]. Following the labeling of the isolated proteins such as with fluorescent cyanine dyes [120], separation in the first dimension occurs within a pH gradient according to the proteins’ isoelectric point. Typically, customizable strips with an immobilized pH gradient (which may vary according to proteins to be separated) are used, and proteins are denatured. The resulting developed gel strip is exposed to sodium dodecyl sulfate to mask the inner charge of the proteins and to assure a separation in the second dimension only according to charge. The second-dimension separates proteins by their mass. This is done typically by SDS-PAGE, and precast gels may likewise be employed.

Finally, the detection of proteins is carried out by a fluorescence reader that manages to interpret the signals from the various fluorescence dyes without overlap. Several thousand protein spots can be separated on a gel [121], which can later be selected and investigated for their structure, such as by matrix-assisted laser desorption ionization time-of-flight (MALDI-TOF). Finally, sequences are aligned by software such as MASCOT or Proteome Discoverer, by comparing their peptide sequences against known databases (e.g., SWISS-PROT). For statistical evaluation, a predefined abundance variation threshold such as 1.5-fold may be defined, and significance be tested by *t*-tests and/or ANOVA (Fisher-protected least significant difference test). For further in-depth information, the reader is referred to more comprehensive reviews [[122], [123], [124]].

#### Quantitative gel-free proteomics.

Nano-flow LC coupled to tandem MS has been used in proteomics for more than two decades to efficiently resolve proteins prior to their analysis [125,126]. In MS-based quantitative proteomics, the relative abundance of proteins is assessed [127,128]. Several methods exist for relative quantification through MS. The most common method is relative stable isotope labeling of proteins. Labeling can be achieved by introducing heavy isotopes of C, H, O, and N into proteins via metabolic labeling techniques, such as stable isotope labeling in mammals (SILAM) [129] and stable isotope labeling by amino acids in cell culture (SILAC) [130,131]. Heavy isotopes can also be introduced by chemical labeling techniques, including isotope-coded affinity tags (ICATs) [130], dimethyl labeling [132]; or isobaric mass tags [133], including but not limited to tandem mass tags (TMTs) [134] and isobaric tags for relative and absolute quantitation (iTRAQ) [10, 135, 136].

Due to their multiplexing capabilities and better protein coverage, iTRAQ and TMT reagents have been increasingly used in plasma proteome studies [10,133,135]. Although these two commercially available tags are widely used in quantitative proteomics, limitations exist, such as cost, fragmentation efficiency, the number of peptides identified, and quantification [[137], [138], [139]]. Label-free approaches also exist; however, they possess major limitations (i.e., lower signal-to-noise ratios and sensitivity). As such, they cannot identify and quantify peptides as isobaric-labeling methods since each sample runs separately, and accordingly, they can potentially offer less precision [140, 141]. For in-depth knowledge of isobaric labeling and its applications, readers are referred to a recently published review article [142].

#### Assay-based methods

Multiplex-based assays are suitable for clinical proteome analysis and are mainly used as an alternative to mass spectrometry, protein pathway array, and next-generation tissue microarrays [143]. Multiplex assays include Luminex, Meso-scale Discovery assay, Single Molecule Array (Simoa), Slow Off-rate Modified Aptamer (SOMAmer) reagent-based (SomaScan) and are used in clinical applications [144,145]. Each multiplex bead- or aptamer-based assay has variations in its sensitivity and specificity [146,147].

To the best of our knowledge, the only multiplex assay used *in vivo* in the context of carotenoids and health is SomaScan [54], which is an aptamer-based, multiplexed, high-throughput, sensitive, and quantitative proteomics assay that consists of single-stranded oligonucleotides embedded on a chip that can bind to protein targets, forming complexes [148]. The method follows standard DNA measuring approaches such as microarrays. This multiplexed technology can simultaneously detect a large number of native proteins (>7000) in biological tissues or extracts [149]. Therefore, this technology allows for high-throughput, untargeted analysis over a wide protein concentration range (10-log range). When comparing SomaScan measured protein abundance of 63 proteins from two human studies with a more classical approach (multiplex immunoassays), Spearman correlation coefficients showed a very large range from poor to high correlations (−0.13 to 0.97 [median 0.5]) [150] for the various proteins, emphasizing that different techniques and platforms can result in rather different findings regarding protein expression. Therefore, it is important to select the technique and evaluate the results for certain individual proteins carefully, preferably employing rather widely accepted standard technology.

### Recent applications

#### Gel-based methods

##### 2D-DIGE

2D-DIGE was used in a few *in vitro* studies to determine the proteomic response in Caco-2 cell lines as a model for inflammatory bowel diseases (IBDs). To investigate the effect of β-carotene and lycopene on inflammation, following TNF-α, IL-1β treatment to induce inflammation, physiological levels (10–25 μg/mL) of these carotenoids were delivered in artificial micelles to Caco-2 cells [56]. Even though the results of the selected biomarkers suggested that isolated carotenoids had no statistically significant anti-inflammatory effect (e.g., on IL-8, NO, and cyclo-oxygenase-2 [COX-2] [through PGF-2α], or regulated NF-κB and MAPK pathways), the proteome analysis revealed that 15 proteins were significantly differentially regulated due to the carotenoid treatments compared with micelles without carotenoids. Most of these proteins were intracellular proteins, 34% of them were involved in metabolic pathways, and 40% were stress response related. β-Carotene and lycopene exposure modulated eleven proteins and one protein (profilin), respectively (Table 2). Between the 2 concentrations of β-carotene, the intermediate concentration (10 μg/mL) downregulated GST, potentially related to oxidative stress and, therefore, inflammation [56].

**TABLE 2:** Recent studies using proteomic techniques highlighting the beneficial role of carotenoids and their potential impact on relevant health outcomes

Study [reference] and main findings	Model	Technique	Design (approach)		Carotenoid	Dosage (duration)	Tissue	Disease state
***In vitro* studies**								
Proteomic response of inflammatory stimulated intestinal epithelial cells to *in vitro* digested plums and cabbages rich in carotenoids and polyphenols [55]	Caco-2 monolayer; Caco-2/HT-29-MTX (90:10 v/v) with THP-1 macrophages	2D-DIGE	N/A (Untargeted)		Derived from food source	Italian plum (carotenoid content: 1.9 mg/100 g) Kale (carotenoid content: 13.3 mg/100 g) (time of cellular exposure-18h)	Model of intestine	Model of IBD

**Findings**	Monoculture: 68 protein-spots were regulated due to the Kale and Italian plum digesta Coculture: 206 protein-spots were regulated due to the Kale and Italian plum digesta Some of the identified proteins included antioxidant enzymes such as catalase, superoxide dismutase and glutathione-S-transferases							

Carotenoid exposure of Caco-2 intestinal epithelial cells did not affect selected inflammatory markers but altered their proteomic response [56]	Caco-2	2D-DIGE	N/A (Untargeted)		Lycopene, β-carotene	10 to 25 μg/mL (time of cellular exposure-4h).	Model of intestine	Model of IBD

**Findings**	β-carotene regulated 15 proteins (involved in antioxidant mechanisms, such as GSTA1) Lycopene regulated 1 protein (profilin-1)							

Unraveling the neuroprotective mechanisms of carotenes in differentiated human neural cells: biochemical and proteomic approaches [151]	SH-SY5Y human neuroblastoma cells	LC-MS/MS (Q-TOF LC/MS)	N/A (Targeted)		Total carotene extract (TMC): α-carotene, β-carotene, γ-carotene, lycopene	0.1 μg/mL (24 h)	Bone Marrow	Oxidative stress

**Findings**	Total mixed carotenoid treatment modulated: RPS: ↓ RPS9, RPS16, RPS4X, RPS19 Tubulins: ↑ TUBA1B, TUBB3, TUBB4B, TUBB6, TUBB2B PDI: P4HB (PDIA1), PDIA3, PDIA6 HSP: HSPA9 (HSP70), HSPD1 (HSP60) and HSP90AB1 (HSP90β), ↓ ROS, ↑ SOD, ↑ CAT							

Systematic investigation of lycopene effects in LNCaP cells by use of novel large-scale proteomic analysis software [152]	LNCaP cells	ICAT ESI-MS/MS and a linear ion trap-FT ion cyclotron resonance mass spectrometer		N/A (Targeted)	Lycopene	0.2 μM (48 h)	Prostate	Chemo-prevention

**Findings**	Lycopene upregulated a group of enzymes associated with the antioxidant response element, involved in detoxification of ROS, including EPHX1, SOD-1, CAT, and TF							

***In Vivo* Animal Studies**								
Torularhodin alleviates hepatic dyslipidemia and inflammations in high-fat diet-induced obese mice via PPARα [153]	Male C57BL/6J mice	HPLC/MS (TMT-labeled peptides)	3 groups (*n*=10/group): 1st group: Control 2nd group: HFD (45% high fat diet) 3rd group: HFD-torularhodin (Targeted)		Torularhodin	40 mg/kg/d in diet (12 wk)	Liver/ adipose tissue	Hepatic dyslipidemia and obesity

**Findings**	Torularhodin: ↑ anti-inflammation proteins (FAS, BAX, ICAM1, OCLN, GSTP1, FAF1, LRP1, APEX1, ROCK1, MANF, STAT3, and INSR) ↓ proinflammatory proteins (OPTN, PTK2B, FADD, MIF, CASP3, YAP1, DNM1L, and NAMPT)							

Proteomic responses of carotenoid and retinol administration to Mongolian gerbils [11]	Gerbils (*n*=30)	2D-DIGE LC-MS/MS	1st group (*n*=6) all-trans β-carotene 2nd group (*n*=6) lycopene 3rd group (*n*=6) lutein 4th group (*n*=6) retinol 5th group (*n*=6) vehicle (control)(Untargeted)		β-Carotene, lutein, lycopene, retinol	Single dose of 100 mg/kg bw (retinol: 53 mg/kg bw) given by oral gavage (12 h)	Liver, white adipose tissue (WAT), plasma	Proteomic response

**Findings**	Carotenoids regulated: 29 protein types in the liver, in the field of protein metabolism, lipid metabolism, detoxification, transport, and energy metabolism. 19 protein types in WAT, involved in cell structure, energy metabolism and lipid metabolism. 66 protein types in plasma, involved in the immune system/inflammation and protein metabolism. Carotenoid administration affected cell structure (22), protein metabolism (15), and immune system/inflammation (19) Similar proteins differentially regulated by lycopene compared to retinol							

***In vivo* human studies**								
Plasma Proteins associated with circulating carotenoids in Nepalese school-aged children [10]	6-8 y old Nepalese children	iTRAQ	500 samples randomly chosen from a 5-arm antenatal micronutrient supplementation trial of 6–8 y old Nepalese children [247] (Untargeted)		β-Carotene, lutein and zeaxanthin, β-cryptoxanthin, α-carotene, and lycopene	Samples from a 5-arm antenatal micronutrient supplementation trial [247]	Plasma	Vitamin A deficiency

**Findings**	4 plasma proteins were associated with β-carotene 11 plasma proteins were associated with lutein/zeaxanthin 51 plasma proteins were associated with β-cryptoxanthin No protein biomarkers associated with α-carotene or lycopene Plasma proteins were notably involved in lipid and vitamin A transport, antioxidant function and anti-inflammatory processes							

Plasma proteome fingerprint associated with circulating carotenoids and retinol in older adults [54]	728 adults (≥65)	SomaScan assay	Cross-sectional analysis (Untargeted)		α-Carotene, β-carotene, β-cryptoxanthin, lutein, zeaxanthin, and lycopene	Samples from a population-based study [248]	Plasma	Aging

**Findings**	Plasma proteomic fingerprint associated with elevated circulating carotenoids in older adults were related to: Sirtuin signaling (NAMPT) Inflammation and oxidative stress (CCNB1, SOD2, IL1RAP, CNDP1) Iron metabolism (HAMP, ferritin) Proteostasis (CLU, CTSV, ACY1) Innate immunity (FCN2) Longevity (CRP, GDF15, THBS2)							

Abbreviations: 2D-DIGE, 2-dimensional difference gel electrophoresis; ACY1, aminoacylase 1; APEX1, apurinic/apyrimidinic endonuclease 1; BAX, Bcl-2–associated X; CASP3, cysteine-aspartic acid protease 3; CAT, catalase; CCNB1, cyclin B1; CLU, clusterin; CNDP1, carnosine dipeptidase 1; CRP, C-reactive protein; CTSV, cathepsin V; DNM1L, dynamin-1-like protein; EPHX1, epoxide hydrolase 1; FADD, Fas-associated death domain; FAF1, Fas associated factor 1; FCN2, ficolin-2; GDF15, growth/differentiation factor 15; GST, glutathione S-transferase; HAMP, hepcidin antimicrobial peptide; HFD, high fat diet; HSP, heat shock protein; IBD, inflammatory bowel disease; ICMA1, intercellular adhesion molecule 1; IL1RAP, interleukin-1 receptor accessory protein; INSR, insulin receptor; LC, liquid chromatography; LRP1, LDL receptor related protein 1; MANF, mesencephalic astrocyte-derived neurotrophic factor; MIF, macrophage migration inhibitory factor; MS, mass spectrometry; NAMPT, nicotinamide phosphoribosyltransferase; OCLN, occludin; PDI, protein disulfide isomerases; PTK2B, protein tyrosine kinase 2-beta; Q-TOF, quadrupole-time of flight; ROCK1, Rho-associated protein kinase 1; RPS, ribosomal protein; SOD, superoxide dismutase; TF, transferrin; THBS2, thrombospondin-2; YAP1, Yes-associated protein 1.

Another *in vitro* study used Caco-2 monoculture and Caco-2/HT-29-MTX (90:10 v/v) coculture with THP-1 macrophages (triple culture) to mimic intestinal epithelia and immune cells as a model for IBD. Inflammation was induced by TNF-α, IL-1β, and LPS. Both cell cultures were treated with kale and Italian plum digesta (1.9 mg/100 g, kale 13.3 mg/100 g) to observe whether these food sources with high carotenoid content reduced inflammation. In this study, carotenoids were not the only phytochemicals in the treatment, as the food source was also rich in polyphenols. In the monoculture and in the triple culture, 27 and 76 uniquely identified proteins, respectively, were differentially modulated. The number of differentially expressed proteins was higher in the kale group, arguably due to the higher carotenoid content. Antioxidant enzymes such as CAT, GST, and SOD were significantly downregulated. This effect was more pronounced in the triple culture and with kale treatment compared to the control (empty digesta). This suggested that carotenoid exposure via kale and plum reduced oxidative stress and inflammation, whereas the difference between the cultures may be explained by the observed higher cellular uptake of carotenoids of the coculture due to the mucus production of the HT-29 MTX cells [55].

An *in vivo* study was carried out on Mongolian gerbils to understand the effect of a single high dose (100 mg/kg bw) of a carotenoid (β-carotene, lutein, or lycopene) or retinol (53 mg/kg bw) administration [11]. The authors used the 2D-DIGE method for liver and adipose tissue, but for plasma, they used an LC-MS/MS approach to reduce the influence of major abundant albumin and to avoid further prior depletion steps. Retinol and lycopene administration yielded the largest number of modulated proteins. Most of the proteins that were modulated were observed in plasma, followed by liver and then white adipose tissue. The most upregulated proteins in the liver were related to energy, lipid, and protein metabolism, whereas in white adipose tissue these were related to cell structure, lipid, and energy metabolism. In plasma, they observed modulation in a variety of proteins that were involved in the immune system/inflammation, cell structure, protein, and energy metabolism. Plasma proteomics also revealed a significant overlap between lycopene and retinol, specifically in immune system/inflammation and lipid metabolism but not with all-trans β-carotene [11], suggesting some common pathways and metabolic effects between these compounds.

#### Gel-free/MS-based methods.

Mass spectrometry has been widely used for proteomics research. A wide variety of strategies can be used for each sample depending on the instrumentation, labeling, fragmentation methods, and analysis.

##### Quadrupole-time of flight (Q-TOF)

Q-TOF as a high-resolution MS, coupled with LC-MS/MS, was used to better understand the protective mechanism of carotenes against cytotoxicity in the human brain [151]. SH-SY5Y human neural cells were differentiated with 10 μM RA for 6 d. Cell viability, lactate dehydrogenase (to determine cytotoxicity), ROS, antioxidant enzyme markers (SOD, CAT), dopamine, and tyrosine hydroxylase were compared. Treatment groups included control (no treatment), 10 μg/mL of 6-hydroxydopamine (6-OHDA) to induce oxidative stress and neurotoxicity, 0.1 μg/mL total carotenes (TMC) that contained α-, β-, γ-carotenes and lycopene at an undetermined concentration followed by 6-hydroxydopamine (6-OHDA) treatment and control (TMC only), and 0.1 μg/mL Levodopa, a standard drug for Parkinson’s disease followed by 6-OHDA treatment and control (Levodopa only). TMC pretreated cells had higher cell viability, alleviated cytotoxicity by increased activity of SOD and CAT enzymes, and blocked overexpression of α-synuclein. For proteomics analyses, authors used label-free mass spectrometry (Q-TOF LC-MS/MS). The findings revealed significant modulations of ribosomal proteins tubulins, protein disulfide isomerases, and heat shock proteins (Table 2) [151].

##### ICAT

To better understand the effects of lycopene at a cellular level, the LNCaP cell line, derived from a metastatic lymph node of prostate cancer, was used as a model of prostate cancer for proteomic analysis [152]. Androgen-sufficient LNCaP and androgen-depleted LNCaP cells were treated with 0.2 μM lycopene solubilized by liposomes (treatment) or liposomes alone (placebo control). Either membrane, cytosolic, or nuclear proteins were extracted and labeled by ICAT prior to analysis by microcapillary μLC-ESI-MS/MS, using both LCQ-DECA mass spectrometer and then again by a (high resolution) linear ion trap-Fourier transform ion cyclotron resonance mass spectrometer (LTQFT-MS). It was observed that lycopene treatment modulated protein expression mainly in the nucleus. Proteins and enzymes that were related to the detoxification of reactive oxygen species were upregulated, such as epoxide hydrolase 1 (EPHX1), SOD-1, CAT, and transferrin [152].

##### TMT

The protective effect of torularhodin, a fungal C40 carotenoid, on high-fat diet-induced inflammation and hepatic dyslipidemia was investigated in 11-wk-old male C57BL/6J mice [153]. Mice (*n* = 30) were randomly divided into 3 groups and were treated for 12 wk. Treatment arms were control (chow diet), high-fat diet (45% calories from fat), high fat + torularhodin diet (45% fat + 40 mg/kg bw/d torularhodin). At the end of 12 wk, there was a significant difference between the high-fat diet and high-fat + torularhodin diet groups in terms of body weight, serum lipid content, triglycerides, total cholesterol, LDL cholesterol, HDL cholesterol, serum insulin, inflammatory mediators (such as TNF-α, IL-6, IL-1β, and LPS), and fasting blood sugar, even though energy intakes were not significantly different. The authors used TMT labeling on hepatic protein samples followed by HPLC separation and MS analysis using an Orbitrap Fusion Tribrid mass spectrometer. A total of 3012 differentially expressed proteins were identified and quantified in the liver tissue. A number of anti-inflammatory proteins such as fas cell surface death receptor (FAS), BCL2 associated X, apoptosis regulator (BAX), and ICAM1 (Table 2) were upregulated, while proinflammatory proteins such as osteopontin (OPTN), protein tyrosine kinase 2 beta (PTK2B) and FAS-associated death domain protein (FADD) were downregulated, indicating that torularhodin possessed anti-inflammatory properties [153]. KEGG pathway enrichment analysis associated torularhodin with multiple signaling pathways, but the PPARα signaling pathway was the most predominant mechanism for its antihyperlipidemic function, as indicated by upregulation of lipid degradation enzymes and downregulation of lipid uptake and synthesis proteins.

##### iTRAQ

The association between proteomic biomarkers and common plasma carotenoids (α-carotene, β-carotene, lutein/zeaxanthin, β-cryptoxanthin, and lycopene) was investigated in 6 to 8 y old Nepalese children to enable a quick determination method for carotenoid status [10]. Plasma (*n* = 500) samples were randomly selected from a 5-arm antenatal supplementation trial maintaining the balance between treatment groups. Samples were immunoaffinity-depleted of albumin, IgG, IgA, transferrin, haptoglobin, and antitrypsin. Plasma carotenoids were measured by HPLC, and proteomic analysis was executed using iTRAQ isobaric mass tags and MS/MS LTQ Orbitrap Velos mass spectrometer. Linear mixed effect models were used to find the correlation between plasma concentration of carotenoids and protein relative abundance. In total, 982 proteins were detected in more than 10% of all samples; 66 of these differentially expressed proteins could be associated with carotenoids. Four were correlated with plasma β-carotene, 11 with lutein/zeaxanthin, and 51 with β-cryptoxanthin. APOA1 (HDL component in plasma) showed a positive correlation with each of these carotenoids. No such correlation was determined for α-carotene and lycopene proteomes [10].

#### Assay-based methods

##### SomaScan

Similar to Eroglu et al. [10], Yamaguchi et al. [54] used proteomic analysis to determine the correlation between plasma proteins and circulating carotenoid and retinol levels in adults (*n* = 728) >65 y, living in Tuscany, Italy, as part of the InCHIANTI study. Plasma carotenoids (α-carotene, β-carotene, β-cryptoxanthin, lutein, zeaxanthin, and lycopene) were measured by HPLC, and proteomic analysis was performed by the SomaScan assay to determine 1301 plasma proteins. Eighty of these proteins correlated positively and 59 plasma proteins negatively with carotenoids. The protein fingerprint of each carotenoid was unique; only 5 proteins were associated with more than one carotenoid. Ferritin was negatively correlated with lutein, zeaxanthin, and lycopene. Four proteins (6-phosphogluconate dehydrogenase (decarboxylating), hepcidin, thrombospondin-2, and choline/ethanolamine kinase) were associated with two carotenoids each. Plasma β-carotene was associated with 85 proteins. β-Cryptoxanthin, lutein, zeaxanthin, and lycopene were associated with 39, 4, 2, and 5 plasma proteins, respectively. α-Carotene and retinol were not associated with any protein. Elevated serum carotene levels were associated with proteins that were related to sirtuins (nicotinamide phosphoribosyltransferase [NAMPT]), cyclin B1 (CCNB1)), inflammation (IL1RAP), oxidative stress (CCNB1, SOD2, CNDP1), iron metabolism (hepcidin antimicrobial peptide [HAMP], ferritin), proteostasis (clusterin [CLU], cathepsin 5 [CTSV], aminoacyclase 1 [ACY1]), innate immunity (ficolin-2), and mortality (C-reactive protein [CRP], growth differentiation factor 15 [GDF15], thrombospondin 2 [THBS2]) [54].

Overall, the results allow deeper insights into the mechanistic effects of carotenoids, and even if the physiological aspects are not fully comprehended, such studies allow comparisons across carotenoids to elucidate differences and commonalities regarding their biological effects, even when studying effects that are more acute.

### Present gaps and perspectives

Proteomics has generated interesting insights into the physiological effects of carotenoids, both following *in vitro* exposure studies and *in vivo* observational studies. Human intervention studies that apply proteomics techniques to study the effects of administered carotenoids have, to our knowledge, thus far not been conducted. Likewise, only two animal intervention studies have been conducted that we are aware of (Table 2).

The main obstacles to the increased employment of proteomics studies targeting carotenoid-related health effects are the high costs requiring sophisticated and quite expensive mass spectrometry such as Q-TOF or MALDI-TOF, in addition to having a long experience in this field of studies, which only few research groups have around the world. However, as proteomics is further downstream compared to transcriptomics, results are clearly expected to be of health relevance, even though there is often a lack of the interpretation of protein abundance and physiological function due to a lack of comprehensive databases [154]. Furthermore, unlike transcriptomics, it is impossible to describe the entire proteome of an organism due to variability in tissues, time-dependent variation and generation of isoforms and post-translational modifications, and a large dynamic range, varying from one protein per cell to several million copies [155]. Regarding the activity of carotenoids, at least several plausible mechanisms relating them to health outcomes are rather likely related to minor abundant proteins such as transcription factors and nuclear receptors, which may be more easily overlooked in untargeted proteomics investigations.

A prominent gap in the area of proteomics with respect to carotenoid-related health effects is that apparently no multiomics studies have been attempted, such as relating proteomics to metabolomics findings, perhaps as the combination of transcriptomics and metabolomics is regarded as more complementary, whereas proteomics itself is already further downstream compared to transcriptomics. However, in other, related, research fields, proteomics has been combined with metabolomics. For instance, the accumulation of carotenoids in bananas was studied by a combination of proteomics and metabolomics [156] plus targeted gene-expression analysis. Another study investigated yeast producing astaxanthin with a combination of proteomics and metabolomics [157] to study effects of different carbon sources. Such multiomics approaches incorporating proteomics would be much desirable in intervention studies involving human subjects to reveal more insights into individual carotenoids and their potential health benefits, especially in sight of the prevention of noncommunicable diseases such as anticancer therapies and cancer prevention, for which the benefits of conducting such complex studies would likely outweigh its original costs. Additional developments such as single cell proteomics [155], development of further assay-based methods such as the SomaScan, may result in the further use of proteomics in carotenoid-related health outcomes.

### Effects of carotenoids as assessed by metabolomics and lipidomics

#### Methodological overview

##### Lipidomics

For studying metabolites of carotenoids themselves within lipidomics, several considerations apply, as reviewed previously [15]. Firstly, compared to original carotenoid concentrations, levels of BCO1/2 produced apocarotenoids in tissues and the circulatory system do occur in pM or low nM concentrations rather than in low μM concentrations as their native precursors. This typically requires more sensitive methods than HPLC, i.e., LC-MS/MS. Ionization modes that have been successfully applied to carotenoid research include APCI [158,159] or ESI, which has been reported as being more sensitive [160]. Carotenoids are generally detected in the positive mode [159,161]. Second, breakdown products may be more polar (i.e., retinol has a logP value of 5.7 compared to 17.6 for β-carotene [162]), and different [more polar] extraction methods and purification methods may apply. Potential extraction protocols for either combined or separate extraction of apo-carotenoids were published recently [57]. A list of potential breakdown products/metabolites that have been measured *in vivo* has been published [15,38], with concentrations ranging as low as 0.1 nmol/L, which may often be around the limit of detection even for LC-MS/MS methods [163].

### Recent applications

A few studies have been carried out with respect to the effect of carotenoids on the human metabolome (Table 3); contrarily, many more studies exist in plants. Peng et al. [14] investigated the relation of circulating carotenoids and the risk of breast cancer in a case-control study within the Nurses’ Health Study, with the underlying rationale that studies have proposed that persons with higher circulating carotenoids are less likely to develop breast cancer [164]. Associations between carotenoids and 293 pre-diagnostic plasma metabolites were studied by the least absolute shrinkage and selection operator (LASSO) statistical tool [165], which avoids forward or backward stepwise procedures. In their study, almost 900 case-control pairs were investigated for plasma α-carotene, β-carotene, β-cryptoxanthin, lutein/zeaxanthin, and lycopene by HPLC. For metabolomics analyses from plasma, samples were prepared by a protein-precipitation step with a mixture of acetonitrile/methanol/formic acid, followed by LC-MS (both positive and negative ionization mode, using an orbitrap MS and a hybrid quadrupole orbitrap MS). More specifically, both hydrophilic and lipophilic compounds were investigated by a targeted approach by comparing results to commercial reference samples. Internal standards were employed to control for analytical variations added during sample extraction. Peak areas were used as quantitative outcomes. Log-transformation and z-score calculation of metabolites was required to obtain normal distributions. Another technique employed, with potential value for all omics techniques that include collecting large datasets, was the imputation of missing values, i.e., if ≤25% of the data were missing, these were assigned half the minimum value (i.e., limit of detection [LOD]), and metabolites with higher than a 25% missing rate were removed. A more prudent approach for imputation could be by methods that also consider an underlying distribution. Such methods could include, for values below the LOD, quantile regression imputation of left-censored data (QRILC [166]), that may need a first log-transformation step, and for data above the LOD the chained equation approach, available, e.g., in the R-package MICE [167]. However, in this study, it was found that metabolites related to immune regulation, such as tryptophan, redox homeostasis (such as glutamine), and also epigenetic regulations (acetylated/methylated metabolites), as well as those involved in β-oxidation, were associated with carotenoid signatures, pointing to the involvement of carotenoids in many biological functions. Especially, β-carotene was associated with a large number of differentially abundant metabolites (*n* = 110), though many metabolites overlapped with several carotenoids (especially provitamin A carotenoids), indicating a somewhat comparable response by various carotenoids. Non-provitamin A carotenoids exhibited more distinct signatures with less overlap, perhaps reflecting a more unique implication in metabolic processes.

**TABLE 3:** Recent studies using metabolomics and lipidomic techniques highlighting the beneficial role of carotenoids and their potential impact on relevant health outcomes

Study [reference] and main findings	Model	Technique	Design (approach)	Carotenoid	Dosage (duration)	Tissue	Disease state
***In vivo* animal studies**							
Ameliorative effect of spinach on nonalcoholic fatty liver disease induced in rats by a high-fat diet [95]	Sprague-Dawley rats (*n*=44)	LC-MS	6 groups: - 1st group: NC (standard diet, *n*=6) - 2nd group: N2.5 (standard diet + 2.5% spinach, *n*=8) - 3rd group: N5 (standard diet + 5% spinach, *n*=8) - 4th group: HC (high-fat diet, *n*=6) - 5th group: H2.5 (high-fat diet + 2.5% spinach, *n*=8) - 6th group: H5 (high-fat diet + 5% spinach, *n*=8) (Untargeted)	Spinach powder (1750 μg total carotenoid/g of spinach powder) : 228 μg neoxanthin/g 292 μg violaxanthin/g, 944 μg lutein/g 46 μg α-carotene/g 225 μg β-carotene/g	5% spinach in diet = 53–56 μg of total carotenoids/d (5 wk) 2.5% spinach in diet = 20–24 μg of total carotenoids/d (5 wk)	Liver	Nonalcoholic fatty liver disease

**Findings**	Consumption of spinach powder and the accumulation of carotenoids in the liver: ↓ SAFA, ↓ ω-6/ω-3 fatty acid ratio, ↓ cholesterol, ↑ MUFA and PUFA						

Lycopene and tomato powder supplementation similarly inhibit high-fat diet induced obesity, inflammatory response, and associated metabolic disorders [93]	Male C57BL/6J mice (*n*=40)	Enzymatic and colorimetric methods	4 groups (*n*=10/group) - 1st group: Control diet - 2nd group: high fat diet (HFD 45% fat) - 3rd group: HFD + lycopene - 4th group: HFD + tomato powder (TP) (Targeted)	Lycopene and TP (lycopene 214 mg/kg of dry TP)	10 mg/kg diet/d (12 wk)	Liver/ epididymal adipose tissue	Inflammation, obesity

**Findings**	Lycopene and TP supplementation: ↓ serum TAG, ↓ FFA, ↓ 8-iso-prostaglandin GF2α, ↓ NEFA, ↑ glucose homeostasis						

Torularhodin alleviates hepatic dyslipidemia and inflammations in high-fat diet-induced obese mice via PPARα signaling pathway [153]	Male C57BL/6J mice	LC-MS	3 groups (*n*=10/group): - 1^st^ group: control - 2^nd^ group: HFD (45% high fat diet) - 3^rd^ group: HFD-Torularhodin (Untargeted)	Torularhodin	40 mg/kg diet/d (12 wk)	Liver/ adipose tissue	Hepatic dyslipidemia and obesity

**Findings**	Torularhodin modulates phenotype parameters: ↓ TG, ↓ TC, ↓ LDL, ↓ LPS, ↓ body weight, ↑ HDL						

Oral lycopene administration attenuates inflammation and oxidative stress by regulating plasma lipids in rats with lipopolysaccharide-induced epididymitis [249]	Sprague-Dawley rats (*n*=31)	LC-MS, hybrid quadrupole orbitrap	Four groups: - Control (*n*=10) - Oil-control (*n*=10, 5 mL/kg diet/d) Single intraperitoneal injection of lipopolysaccharide in 0.9% sodium chloride, *n*=5 Continuous intragastric lycopene in oil, *n*=6 (Untargeted)	Lycopene	5 mg/kg bw/d (4 wk)	Plasma; Cauda epididymis (male reproductive system)	Epididymitis

**Findings**	Lycopene: ↑ PC, ↓ TAG, ↓ DAG, ↓ phosphatidylethanolamine						

Integrated metabolomics, lipidomics, and genomics reveal the presence of a new biomarker, butanediol glucuronide, associated with the activation of liver ketogenesis and lipid oxidation by tomato-based sofrito in obese rats [250].	Obese Zucker rats	LC-HRMS	4 groups (*n*=5/group): 1^st^ group: lean rats fed chow diet 2^nd^ group: obese rats fed control chow diet 3^rd^ group: lean rats fed chow diet + sofrito 4^th^ group: obese rats fed chow diet + sofrito (Untargeted)	Sofrito (a tomato and olive oil based sauce with high content of carotenoids)	2% w/w supplemented chow diet ad libitum (8 wk)	Adipose tissue, epididymal and visceral	Obesity

**Findings**	In groups supplemented with sofrito: ↑ expression of esterification enzymes mediating the synthesis of TG from DG, DGAT1 and DGAT2, ↑ expression of CPT1A and PRDM16, ↑ expression of HMGCoA and PKL						

Orally administered crocin protects against cerebral ischemia/reperfusion injury through the metabolic transformation of crocetin by gut microbiota [251]	Male specific-pathogen-free Sprague-Dawley rats	GC-MS	2 sham-operated groups and 2 model-operated groups were separately: Intravenous administration: Positive control group: Edaravone Injection (3 mg/kg bw). Model group: Crocin (1 mg/kg bw) Oral administration: Positive control group: free access to food and water. Model group: Crocin (60 mg/kg bw) (Untargeted)	Crocetin	Intravenous administration: 1 mg/kg bw or 60 mg/kg bw (2 h after the onset of ischemia) Oral administration prior to and on the same day of the MCAO procedure (4 d)	Rat transient middle cerebral artery occlusion (MCAO) model	Cerebral ischemic/reperfusion (I/R) injury

**Findings**	Gut flora plays a key role in the transformation of crocin into crocetin and is the potential target for the cerebral-protection of crocin in MCAO model rats Oral administration of crocin ameliorated the dysfunctional metabolism of the brain to normal status: ↑ MCAO-protective effects than intravenous administration, ↑ concentration of GABA						

***In vivo* human studies**							
Associations between circulating lipids and fat-soluble vitamins and carotenoids in healthy overweight and obese men [16]	Overweight and obese men with low-grade inflammation (*n*=35)	LC-MS Linear Trap Quadrupole	Data from the placebo arm of a randomized, double-blind, placebo-controlled, 5-wk intervention trial (Untargeted)	α-Carotene, β-carotene, β-cryptoxanthin, and lycopene	Plasma lycopene 0.62 μM, plasma α-carotene 0.06 μM, plasma β-carotene 0.4 μM, plasma β-cryptoxanthin 0.22 μM (5 wk)	Plasma	Inflammation, obesity

**Findings**	Interindividual variability in circulating lycopene was explained by low-abundant TGs with 40–52 carbons => lycopene was inversely related to low-abundant TGs. Interindividual variability in circulating of other carotenoids (α-carotene, β-carotene, and β-cryptoxanthin) was not explained by the evaluation of lipids						

A multi-omic analysis for low bone mineral density in postmenopausal women suggests a relationship between diet, metabolites, and microbiota [213]	Postmenopausal women (*n*=92) (≥45 y old, postmenopausal status (12 consecutive months without menstruation)	Electrospray tandem MS	Postmenopausal women classified into: - Normal bone mineral density (normal-BMD, *n*=34) - Low-BMD (*n*=58) (Untargeted)	Lycopene from diet	Data and samples collected from [252]	Serum; gut microbiota	Bone mineral density

**Findings**	The low-BMD group had fewer observed species, higher abundance of *γ-Proteobacteria*, lower consumption of lycopene, and lower concentrations of leucine, valine, and tyrosine compared with the normal-BMD group. Leucine, valine, and tyrosine correlated positively with the abundance of *Bacteroides*.						

Abbreviations: bw, body weight; CPT1A, carnitine palmitoyltransferase 1A; DAG, diacylglycerol; FFA, free fatty acid; DGAT1/2, diglyceride acyltransferase 1/2; GABA, γ-aminobutyric acud; HMGCoA, (3S)-hydroxy-3-methylglutaryl-CoA; HRMS, high-resolution mass spectrometry; LC, liquid chromatography; LPS, lipopolysaccharide; MS, mass spectrometry; NEFA, non-esterified fatty acids; PC, phosphatidylcholine; PE, phosphatidylethanolamine; PKL, liver-type pyruvate kinase; PRDM16, positive regulatory domain containing 16; SAFA, saturated fatty acid; TAG, triacylglycerol; TC, total cholesterol; TG, triglyceride; UPLC, ultra-performance liquid chromatography.

In an animal study, though by the time of writing only available as an abstract by Daniels et al. [168], mice with or without double knockout (DKO) for BCO1/BCO2 receiving a highly carbohydrate-refined diet were investigated for plasma by QTOF-MS, allowing for high-resolution MS. More specifically, for targeted metabolomics, an MxP Quant 500 kit was employed, which allowed for the quantification of 630 metabolites from 26 biochemical classes, together with an untargeted phospholipid investigation. The DKO mice showed significantly elevated levels of MDA, as well as lower levels of adiponectin and hepatic bile salts, and alterations regarding cholesterol, triglycerides, ceramides, and phospholipids were also recognized, pointing out changes linking carotenoid metabolites to levels of oxidative stress and inflammation, perhaps via their influence on transcription factors such as NF-κB and Nrf2.

In a study by Kelly et al. [16], the authors studied the human lipidome (according to lipid class, carbon count, and the number of unsaturated bonds) in relation to the interindividual variability of plasma concentrations of retinol, carotenoids, 25-hydroxyvitamin D3, α-tocopherol, γ-tocopherol, and phylloquinone in 35 adult overweight men. The within-person variability of lycopene levels was associated with low-abundance triglycerides, while other carotenoid concentrations were not associated with lipids. A partial least square regression model was applied to avoid overfitting to the small number of subjects. In this study, carotenoids were determined by HPLC-UV-Vis, while lipids were assessed by LC-MS. The study is an interesting example of a small-scale hypothesis-generating approach with a low number of subjects.

### Present gaps and perspectives

A few studies have been conducted that explore the relation between carotenoids and health outcomes by means of metabolomics. Their number, however, is still small when compared to those investigating carotenoids in plants, where a number of studies have explored lipidomics with a focus on carotenoids and metabolites. Regarding lipidomics in human tissues, applications of metabolomics are impeded by the low levels of circulating carotenoids and their metabolites, often in the subnanomolar range. In this respect, purification, and concentration, such as by solid phase extraction (e.g., reverse-phase [RP]-18 or RP-30) [169] or liquid-liquid extraction, may help, though at risk of further discrimination.

In addition, there is a lack of commercially available standards except for native carotenoids and a few selected metabolites, such as retinoids. This is often required, as many carotenoids and breakdown products have the same nominal mass, i.e., they cannot be separated even by high-resolution MS. Improved separation techniques such as RP-30 phases, offering a better timely resolution of carotenoid isomers and possibly longer apo-carotenoids, may help to separate compounds in time [15]. GC-MS may offer more resolution power with time (i.e., number of theoretical plates), but can only be used for stable and volatile, i.e., shorter carotenoid metabolites. For instance, Aust et al. [170] determined the lycopene oxidation product 2,7,11 trimethyltetradecahexene-1,14-dial by GC-MS.

Regarding LC-MS, new powerful tools have become more available, such as LC-MS-MS, e.g., by triple-quad technology to allow more detailed insights into fragmentation patterns of metabolites, or Orbitrap-technology for high resolution (100,000 or more), allowing exact mass determination and thus more accurately determining molecular formulas. Novel separation techniques such as 2D-LC, which allow online second separation of a peak following a first separation on another stationary phase, may offer the potential to better separate lipid molecules, including carotenoids and metabolites, and has been proposed for carotenoid analysis [159]. Other detectors may offer additional options, such as fluorescence detection for phytoene/phytofluene or the use of electrochemical detection for some compounds, such as being used for polyphenols [171], which is more sensitive than UV-Vis, at least for less UV-Vis active compounds such as carotenoid metabolites. Cacciola et al. [172] employed a set-up of normal phase column (cyano-column) combined with an RP-18 column followed by mass spectrometry for the separation of paprika carotenoids. New detectors such as HPLC-NMR [173] may provide additional structural information compared to LC or MS alone. Even though some NMR applications have demonstrated its usefulness down to amounts down to 1.4 nmol (800 ng) for carotenoids, a limitation is still its sensitivity compared to, e.g., LC-MS-MS technology.

When conducting human studies, of course, another challenge, even in controlled intervention studies, are confounding factors or covariates, such as fruit and vegetable intake, polyphenols, or dietary fiber, which are also abundant in carotenoid-rich sources. Thus, these aspects should likewise be considered to strengthen the evidence of carotenoids as causal agents impacting health-relevant outcomes, and meaningful selective regression tools such as LASSO may aid in this approach.

However, due to the involvement of so many biological pathways, a multitude of changes in the metabolomic signature are expected, and the higher availability of sensitive and selective methods to detect a large number of compounds at the same time makes the employment of metabolomics and lipidomics in carotenoid research more likely and more rewarding.

## Carotenoids within Gut Microbial Communities

### Methodological overview

#### 16S rRNA sequencing

Current approaches to analyze microbial communities in a given ecosystem include both 16S ribosomal rRNA (a marker gene, allowing classification on the genus/phylum level) sequencing or shotgun metagenomics [174,175]. Surveying the gut microbiome through these sequencing methods offers multiple advantages compared to culture-dependent methods, such as not requiring the bacteria in a given ecosystem to be culturable, and can provide a relative abundance of all bacteria and simultaneous sequencing of many samples [176]. Sequence-based analyses of microbial communities are becoming readily available, are sensitive and cost-efficient due to significant advancements in next-generation sequencing (NGS) platforms [177,178]. Among them, 16S rRNA sequencing, also referred to as amplicon sequencing, can be used for taxonomic classification [179]. The 16S rRNA gene is highly conserved, containing 9 hypervariable regions (V1 to V9) with nucleotide differences that can be used to identify bacteria in a given community [180,181].

DNA sequences can vary in amplifying primers associated with variable regions; subsequently, primers cannot anneal to the same extent, leading to bias in PCR amplification [182]. More recently, full-length 16S rRNA sequencing has been applied to overcome challenges observed in short-read sequencing of variable regions, such as the underrepresentation of *Bifidobacterium* due to primer-associated bias [183]. Full-length 16S rRNA sequencing can also offer better discriminatory power to other certain bacterial taxa, including *Clostridium*, *Staphylococcus*, and *Enterococcus*, compared to short-read sequencing [183]. However, taxonomic resolution is still limited to the genus level at best, even after mitigating these limitations, and functional information derived from marker gene sequencing is limited [184,185]. 16S rRNA sequencing has been used to analyze microbiomes in various ecosystems, including soil, water, and the mammalian gut. It also is used to provide an understanding of various chronic diseases, including obesity [186], IBD [187], diabetes [188], and also gastrointestinal cancers [189], all of which having been associated with carotenoid intake.

#### Metagenomics

Sequencing all microbial genomes (i.e., shotgun metagenomics), including viral and eukaryotic organisms, can be used to obtain a genomic-level understanding of the microbiome. Whole-genome shotgun sequencing is the method of choice if the research project aims to determine the presence of all microorganisms and their genomic content [190], providing better taxonomic resolution [191,192]. Metagenomics can also be used to provide the functional capacity of microbial communities [193]. Although there are several strengths of profiling microbial communities via shotgun metagenomics, a few limitations exist. It is relatively expensive, and sample preparation is not as straightforward as 16S rRNA sequencing. Moreover, host genetic materials need to be removed prior to analysis. To remove host-related reads, several bioinformatic pipelines, including Bowtie2 [194], Trimmomatic [195], Burrows-Wheeler Transform (BWT) [196], and DeconSeq [197] are employed. For a comprehensive review on shotgun metagenomics, we direct the reader to a recently published review article [198].

#### Metatranscriptomics

To aid in high-resolution analysis of microbial communities, metagenomics data can be integrated with metatranscriptomics. It profiles the microbial community transcripts, providing information on gene expression. Although metagenomics can provide the potential functional output of microbial communities, metatranscriptomics can provide functional information since the presence of DNA or gene itself does not necessarily mean that it is expressed. Also, transcriptomes vary more within individuals than metagenomes [199]. Metatranscriptomics provided microbial community responses to perturbations such as xenobiotic exposure, highlighting the importance of measuring actual gene expression in microbial communities [200].

Transcriptional pathways by microorganisms revealed that metagenomically abundant microorganisms may be inactive or dormant, yet their transcripts can be either more active or only detectable at the RNA level and, accordingly, can change host physiology [201,202]. These studies underscored the importance of sequencing RNA and surveying the transcriptional activity of gut bacteria, providing new insights into the mechanism of gut dysbiosis, i.e., an altered configuration of microbiota associated disease. Gut dysbiosis may also be a target for carotenoid intervention, as some studies have proposed beneficial effects of carotenoid intake on gut microbiome diversity [21], or more general gut health, possibly related to reduced local oxidative stress [45] or, for provitamin A carotenoids, secretion of IgA [203]. Although utilizing metatranscriptomics will be useful in understanding how changes in microbial gene expression can be linked to host biology, there are several drawbacks. One of these is that metatranscriptomics can only be used in intact RNA during extraction; another notable limitation is the fact that it is mainly used in stool samples due to the presence of a high proportion of host genetic material in biopsy samples. The depletion of the host genome can be achieved in multiple ways. Several of them are included; via enrichment of microbiome [204, 205], through removing polyadenylated transcripts (polyA depletion) [206], or *in* *silico* removal of rRNA and host RNA by means of bioinformatic pipelines, such as Bowtie2 [194] or SortMeRNA [207]. For a comprehensive review on metatranscriptomics, we direct the reader to a recently published review article [208].

### Recent applications

16S rRNA sequencing is the most commonly applied method for investigating the effects of carotenoids on the gut microbiome. Astaxanthin treatment (50 mg/kg bw/d for 12 wk) on a high-fat (35% kcal from fat) and ethanol-fed diet led to gut microbiota changes that correlated with alcoholic fatty liver disease alleviation in C57BL/6J mice (Table 4). Such astaxanthin administration led to decreases in *Bacteroidetes*, *Proteobacteria*, *Butyricimonas*, *Bilophila*, and *Parabacteroides* populations as well as increases in *Verrucomicrobia* and *Akkermansia* populations compared to the ethanol-fed group [209]. Astaxanthin (0.04% wt/wt in feed for 8 wk) was also administered to wild-type and BCO2^−/−^ C57BL/6J mice, which led to differential changes in liver inflammation and gut microbiota. A 385% increase of *Akkermansia muciniphila* was detected in the gut of BCO2^−/−^ mice compared to wild-type, which could be explained by there being 10-fold more astaxanthin present in the liver due to the lack of BCO2-induced cleavage [210].

**TABLE 4:** Recent studies using microbial profiling techniques highlighting the beneficial role of carotenoids and their potential impact on relevant health outcomes

Study [reference] and main findings	Model	Technique	Design (approach)	Carotenoid	Dosage (duration)	Tissue	Disease state
***In vivo* animal studies**							
Gut microbiota regulation and anti-inflammatory effect of β-carotene in dextran sulfate sodium-stimulated ulcerative colitis in rats [212]	Specific pathogen-free male SD rats	16S rRNA	4 groups (*n*=6/group) - 1st group: Normal diet (control group) - 2nd group: β-carotene supplementation - 3rd group: Dextran sulfate sodium (DSS), ulcerative colitis model - 4th group: DSS and β-carotene (Untargeted)	β-Carotene	50 mg/kg bw (1 wk)	Gut microbiota	Ulcerative colitis, inflammation

**Findings**	β-Carotene: ↑ abundance of *Faecalibacterium*, *Firmicutes* and *Actinobacteria; ↓ Bacteroidetes and Proteobacteria*						

Alteration of fecal microbiota by fucoxanthin results in prevention of colorectal cancer in AOM/DSS mice [211]	ICR mice (males, 5-wk-old)	16S rRNA	4 groups (*n*=5/group). - Groups 1 & 2 (azoxymethane [AOM]/DSS-treated mice): single intraperitoneal injection of AOM (10 mg/kg BW) + 3.0 w/v% DSS in drinking water for 1 wk - Groups 3 & 4: saline injection + water for 1 wk - Groups 1 & 3: oral administration of Fucoxanthin-oil, 3× per week during 14 wk - Groups 2 & 4: oral administration of oil, 3× per week during 14 wk (Untargeted)	Fucoxanthin	30 mg/kg bw, 3× per week (14 wk)	Gut microbiota	Inflammation-associated colorectal cancer

**Findings**	Fucoxanthin: ↓ *Bacteroidlales and Rikenellaceae* vs. AOM/DSS control mice, ↑ Number of apoptosis-like cleaved caspase-3 cells in both colonic adenocarcinoma and mucosal crypts in group 1 vs. group 2, ↑ *Lachnospiraceae* vs. AOM/DSS control mice						

Astaxanthin-shifted gut microbiota is associated with inflammation and metabolic homeostasis in mice [210]	C57BL/6J mice (*n*=80)	16S rRNA	C57BL/6J mice of each sex grouped into 4 treatments (8 treatment groups in total considering both sexes (*n*=10 mice/group) - KO-CONT (BCO2 KO mice fed control) ×2 - KO-ASTX (BCO2 KO fed astaxanthin) ×2 - WT-CONT (WT fed control) ×2 - WT-ASTX (WT fed astaxanthin) ×2 (Untargeted)	Astaxanthin	0.04% (wt/wt) in diet (8 wk)	Gut microbiota	Inflammation, oxidative stress, and metabolic homeostasis

**Findings**	BCO2 KO mice fed astaxanthin had 10-fold more astaxanthin than WT mice fed astaxanthin in liver, resulting in: ↑ 385% of gut *Akkermansia muciniphila* in male KO mice than the WT mice						

Astaxanthin prevents alcoholic fatty liver disease by modulating mouse gut microbiota [209]	C57BL/6J mice (n=60)	16S rRNA	Five groups (n=12 mice/group): - 1st group: normal standard growth diet for 16 wk. - 2nd group: high-fat liquid diets (HFT, 35% fat, 18% protein, 47% carbohydrates) for 16 wk. - 3rd group: HFT for 2 wk acclimation, then HFT combined with the astaxanthin (AST group, 50 mg/kg bw) treatments for 12 wk. - 4th group: HFT for 2 wk acclimation, then HFT combined with ethanol-containing (Et group, 5% ethanol v/v, for 36% of the total caloric intake) treatments for 12 wk. - 5th group: HFT for 2 wk acclimation, then HFT combined with ethanol plus astaxanthin (EtAST group) treatments for 12 wk (Untargeted)	Astaxanthin	50 mg/kg bw (12 wk)	Liver	Alcoholic fatty liver disease

**Findings**	Astaxanthin: ↓ *Bacteroidetes, Proteobacteria, Butyricimonas, Bilophila*, and *Parabacteroides* compared to ethanol group, ↑ *Verrucomicrobia* and *Akkermansia* compared to ethanol group						

***In vivo* human studies**							
A multi-omic analysis for low bone mineral density in postmenopausal women suggests a relationship between diet, metabolites, and microbiota [213]	Postmenopausal women (*n*=92) (≥45 y, postmenopausal status [12 consecutive months without menstruation])	16S rRNA	Postmenopausal women classified into: - Normal bone mineral density (normal-BMD, *n*=34) - Low-BMD (*n*=58) (Untargeted)	Lycopene	Data and samples collected from [252]	Gut microbiota	Bone mineral density

**Findings**	Lycopene consumption positively correlated with *Oscillospira* and negatively correlated with *Pantoea* genus abundance The low-BMD group had lower consumption of lycopene, and higher abundance of *γ-Proteobacteria*, compared with the normal-BMD group. Intestinal microbiota of women with vitamin D deficiency was related to *Erysipelotrichaceae* and *Veillonellaceae* abundance compared to the vitamin D nondeficient group.						

Dietary and plasma carotenoids are positively associated with alpha diversity in the fecal microbiota of pregnant women [253]	Pregnant women (*n*=27)	16S rRNA	2-arm, randomized, controlled gestational study with pregnant women at 3 different time points: - 1st group: 32-wk gestation, preintervention - 2nd group: 36-wk gestation, mid-intervention - 3rd group: 6 wk after child is born, post-intervention (Untargeted)	Diet containing α- and β-carotene, lutein and zeaxanthin, β-cryptoxanthin, and trans-lycopene	Variable (from 32-wk gestation to 6-wk postpartum).	Fecal microbiota	Microbiota diversity

**Findings**	α-Carotene: ↓ *Akkermansia* and ↑ *Phascolarctobacterium*. β-Carotene: ↑ *Ruminococcaceae UCG002* Trans-lycopene: ↓ *Akkermansia*, ↓ *Escherichia Shigella*, *↓ Phascolarctobacterium*, ↓ *Ruminococcaceae UCG002*, ↓ *Prevotella 9* and ↑ *Ruminococcus 2* β-Cryptoxanthin: ↑ *Phascolarctobacterium* and ↓ *Prevotella 9* Lutein and zeaxanthin: ↑ *Akkermansia*, ↑ *Phascolarctobacterium* and ↓ *Prevotella 9*						

Prebiotic effect of lycopene and dark chocolate on gut microbiome with systemic changes in liver metabolism, skeletal muscles and skin in moderately obese persons [21]	30 volunteers (15 women and 15 men), mean age of 55 ± 5.7 y and with moderate obesity, 30 < BMI < 35 kg/m^2^	16S rRNA	Volunteers randomized into five equal interventional groups: - 1st: 10 dark chocolate (DC) containing 7 mg of lycopene - 2nd: 7 mg lycopene formulated with medium saturated fatty acids (GAL-MSFA) - 3rd: 30 mg GAL-MSFA - 4th: 30 mg lycopene formulated with polyunsaturated fatty acids (GAL-PUFA) - 5th: 10 g DC (Untargeted)	Lycopene	7–30 mg (1 mo)	Fecal microbiota	Obesity

**Findings**	Lycopene groups: ↑ *Bifidobacterium adolescentis* and *Bifidobacterium longum*						

Abbreviations: AOM, azoxymethane; BCO2, β-carotene oxygenase 2; BMD, bone mineral density; bw, body weight; DC, dark chocolate; DSS, dextran sulfate sodium; GAL, GA-lycopene, a proprietory product; HFT, high-fat liquid diet; SD, Sprague-Dawley.

Fucoxanthin (30 mg/kg bw 3× weekly for 14 wk) altered the fecal microbiota of mice afflicted with colorectal cancer induced by azoxymethane (AOM)/dextran sulfate sodium (DSS). This fucoxanthin administration decreased the *Bacteroidales* and *Rikenellaceae* populations, as well as increased the *Lachnospiraceae* population compared to the AOM/DSS control mice [211]. β-Carotene (50 mg/kg bw/d for 7 d) also reversed DSS-induced ulcerative colitis gut microbiota changes in male-specific pathogen-free rats via the increase of *Firmicutes* and *Actinobacteria* relative populations, in addition to the reduction of *Bacteroidetes* (thus reversing the *Firmicutes/Bacteroidetes* ratio) and *Proteobacteria* relative populations. Such β-carotene treatment also led to an increase in butyrate-producing *Faecalibacterium,* which is negatively correlated with IBD through the inhibition of intestinal epithelial NF-κB activation [212].

A human double-blinded trial that provided early evidence regarding the prebiotic effects of lycopene and dark chocolate in moderately obese individuals (*n* = 30, BMI 30–35 kg/m^2^) found that lycopene (7–30 mg/d for 1 mo) increased populations of fecal *Bifidobacterium adolescentis* and *Bifidobacterium longum* [21]. Another study investigated the correlations between diet, microbiota composition, and serum metabolic profile of postmenopausal women (*n* = 92) with low bone mineral density, from the Health Workers Cohort Study, which found that lycopene consumption was positively associated with fecal *Oscillospira* abundance and negatively associated with *Pentoea* abundance [213].

Microbiome studies regarding carotenoid supplementation to animals appeared to be conducted primarily in conventional wild-type models (i.e., C57BL/6J mice) rather than transgenic ones, with the exception of strains related to carotenoid metabolism (i.e., BCO1/2 knockouts), as most diseases (i.e., high-fat diet-induced obesity, ALD, AOM/DSS-induced colitis, etc.) seem to be easily achieved with minimal concern for confounding host organism-induced interference with treatment-induced changes on the microbiota. An exception to this was found in a study involving BCO1/BCO2 DKO mice fed with a high-refined carbohydrate diet (HRCD), which uncovered that BCO1/2 ablation led to decreased alpha diversity compared to the HRCD-fed wild-type mice [168]. According to these microbiota-related studies, carotenoids appear to be capable of modulating the gut microbiome profile and in part, alleviating the gut dysbiosis induced by various disease states. This was demonstrated as carotenoids in multiple cases decreased *Bacteroidetes* and increased *Firmicutes,* thus influencing the pivotal *F/B* ratio involved in gut homeostasis. Furthermore, carotenoids in the studies listed here consistently promoted populations with short-chain fatty acid production i.e., *Akkermansia muciniphila, Oscillospira, Bifidobacterium, Faecalibacterium, Lachnospiraceae* and thus portray probiotic potential, thereby acting as prebiotics.

Although studies listed here have demonstrated that carotenoid administration influences the mouse and human gut microbiota, the existing research remains limited as all used a marker gene sequencing approach only and were limited to changes in microbial composition. Carotenoids comprise a large family of substances, of which lycopene, astaxanthin, and β-carotene are only a small part. *In vitro* experiments have suggested that a simulated gut microflora could degrade carotenoids and generate short-chain fatty acids [214] and vitamin A [215]. These exciting results motivate the study of the *in vivo* mechanisms underlying the response of the gut microbiota to carotenoids, which are still unclear.

Recent studies suggest that the biosynthesis of carotenoids by gut microbiota may help alleviate or prevent certain diseases. For instance, a higher level of phytoene dehydrogenase, related to carotenoid biosynthesis, was observed in the gut microbiota from healthy individuals, as compared to the patients with symptomatic atherosclerotic plaques [216]. Furthermore, gut microbiota from patients with sarcopenia had a lower abundance of α-carotene biosynthesis compared to healthy controls [217]. Similarly, carotenoid biosynthesis in the oral microbiota was associated with protection against esophageal adenocarcinoma [218]. Interestingly, the oral microbiota of sailors was altered during a long sea voyage, reducing the abundance of microbial genes associated with biosynthesis of carotenoids [219]. These studies indicate that environment and health correlate with carotenoid metabolism in the microbiome, motivating further study to determine whether these microbiome changes play a causal role in the etiology of diseases. For more information about molecular-level views of carotenoid-induced gut microbiota changes, the readers are referred to a recent review article published by members of our group [45] as well as other sources [220].

### Present gaps and perspectives

A wide variety of microorganisms produce carotenoids, often giving them a striking color and the ability to resist oxidative stress. The addition of duroquinone, an O_2_ generator, to yeast-malt broth increased total carotenoid content in the yeast *Phaffia rhodozyma*. Moreover, H_2_O_2_ resistance was related to carotenoid levels, indicating an antioxidant role during aging [221]. Another study observed a similar effect of carotenoid antioxidant capacity in *Rhodotorula mucilaginosa* [222]. It has also been reported that deleting the carotenoid biosynthesis gene in *Staphylococcus aureus* caused the mutant to be sensitive to oxidant killing [223], and in microalgae, carotenoids play an essential role in photoprotection [224]. Therefore, carotenoids play critical roles in microbial colonization and enhance the response to stress conditions. The gut microbiota comprises numerous microorganisms, especially bacteria, and the roles of carotenoids in this environment remain to be elucidated because although the photoprotective effect of carotenoids is not likely to be relevant in the gut, their ability to enable microbes to resist oxidative stress might be very important, especially in the context of inflammation. Coupled with the presence of carotenoids and their precursors in the gut from the diet, there is potential for gut microbes to exploit these valuable molecules for survival and manipulation of human health. To investigate this hypothesis further, more work must be carried out to elucidate carotenoid-related pathways and genes in gut microbes.

In addition to metagenomics, metatranscriptomics, as mentioned previously, could reflect gene expression, especially by comparing the differentially expressed genes from different samples. In the future, researchers can measure the expression level of carotenoid-modifying enzymes in the gut microbiota to reveal underlying pathway changes in both mice and humans. This approach has the potential for discovering the mechanisms by which diet- and microbiome-derived carotenoids impact the gut microbiota and the host.

With advances in sequencing platforms, more metagenomes and metatranscriptomes can be sequenced by third-generation sequencers (TGS), such as the PacBio or MinION platform, which produce longer reads compared to next-generation sequencers (NGS) platforms such as Illumina. Combining TGS and NGS can result in higher assembled contig length (n=50) and accuracy. A recent study showed that TGS can assemble much more complete metagenome-assembled genomes, containing more genes of biotechnological interest [225]. Some short-read assemblers, such as SPAdes [226] and Trinity [227], also have already added parameters to handle TGS reads, enabling easy assembly of high-quality genomes and transcriptomes. The application of TGS and NGS in sequencing metagenomes and metatranscriptomes holds promise for identifying carotenoid-related genes and pathways in future research.

Overall, 16S rRNA sequencing, metagenomics, and metatranscriptomics, combined with NGS and TGS, can characterize the taxonomy, gene abundance, and gene expression levels in the gut microbiota. Together, they enable a comprehensive understanding of carotenoid-related changes in the gut microbiota and their biosynthetic pathways. Given the limited research on the carotenoid metabolism within the gut and its relationship to human health, metaomics holds great potential in future carotenoid-related research and is likely to yield promising outcomes.

## Conclusions and Perspectives

The employment of a variety of omics-based techniques, including transcriptomics, proteomics, metabolomics, and metagenomics/metatranscriptomics, has been rapidly increasing in science in recent years. This has included studies on the health benefits of carotenoids, even though compared to studies in plants, the number of studies appears to be far more limited, even though there have been a number of mechanistic studies in cell culture such as Caco2 models, animal models such as rats and gerbils, and also human studies, including intervention trials.

**Figure 2:**
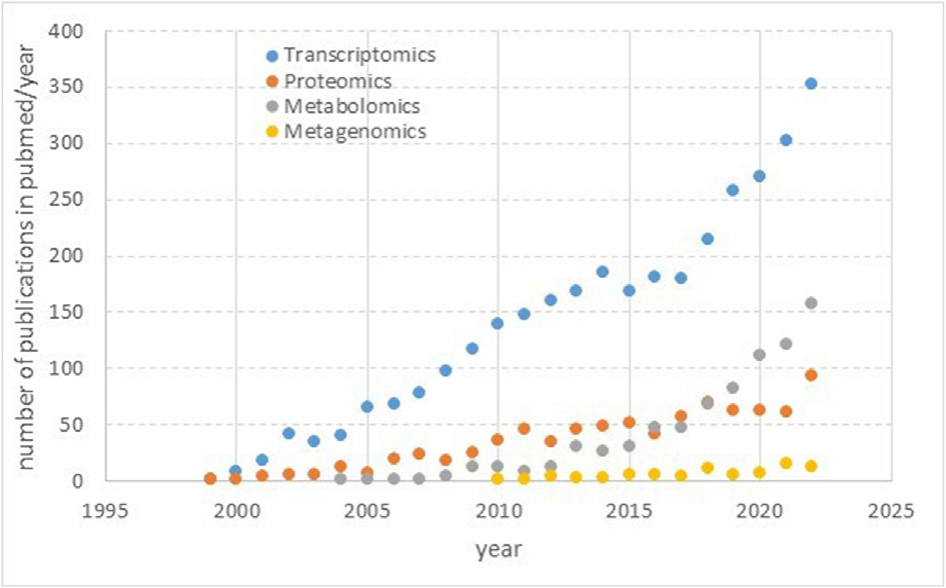
Number of publications listed in PubMed with keywords regarding carotenoids and various omics techniques.

**Figure 3:**
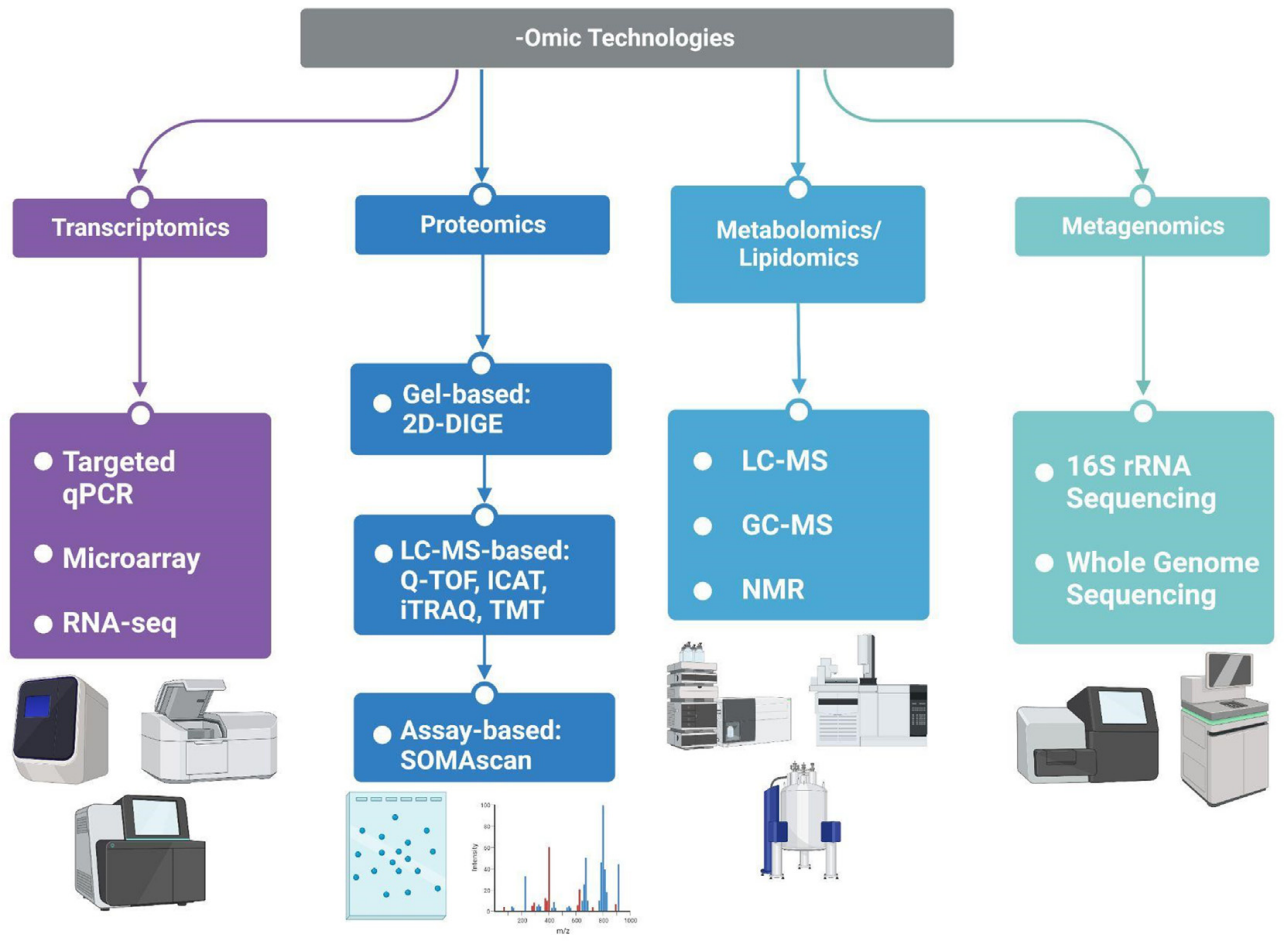
Overview of current -omic technologies, most of which have been used for carotenoids and human health endpoints. Images depict common instrumentation utilized for techniques within their respective categories. Created with BioRender.com 2D-DIGE, 2-dimensional difference gel electrophoresis; GC, gas chromatography; ICAT, isotope-coded affinity tag; iTRAQ, isobaric tags for relative and absolute quantitation; LC, liquid chromatography; MS, mass spectrometry; NMR, nuclear magnetic resonance; Q-TOF, quadrupole-time of flight; TMT, tandem mass tag.

The majority of published studies (Table 1) in the field of carotenoid-related health effects has been clearly conducted on transcriptomics, followed by metabolomics/lipidomics, proteomics, and metagenomics. The higher apparent costs of the latter techniques compared to targeted transcriptomics analyses, combined with their less complex interpretability, i.e., requiring less sophisticated statistical tools, is a likely explanation for this observation. Some techniques have also been available on the market for a longer time, e.g., RT-PCR for several decades (since the mid-90s, with the first publications appearing in 1995 [78] while metatranscriptomics have even developed much more recently, with first publications in 2006 (PubMed, Figure 2). Other omics techniques have been known for some time but have not been very widespread, such as 2D-DIGE, which has been around since 1975, although publications appeared to increase after 1999 (PubMed). However, as seen in Figure 2, while several hundreds of publications have been retrieved for keywords combining carotenoids and omics, very few indeed have dealt with human-relevant health outcomes (see tables). However, because of the wider availability of sophisticated instrumentation, including high-resolution MS (metabolomics, proteomics), the continuously decreasing costs for various sequencing techniques (transcriptomics, metagenomics/metatranscriptomics), and also the larger availability of commercial kits such as DNA microarray (transcriptomics) and SomaScan (proteomics), as well as the increased interest in scrutinizing dietary habits that are helpful in the prevention or even amelioration in chronic diseases, including carotenoids as part of a healthy diet, will likely increase the use of these omics-tools in the near future (Figure 3).

Regarding sampling strategies, a large number of tissues from animals and humans, including adipocytes, liver cells, prostate and lung tissue, placenta, mononuclear blood cells, prostate tissue, bone marrow, and plasma, as well as stool samples have been employed. Urine for metabolomics has been overlooked thus far but is perhaps not the primary target for the lipophilic carotenoids to result in rapidly measurable changes, even though urine is frequently target in general by metabolomics studies [228,229]. For human studies, clearly, plasma and white blood cells have been the preliminary target, in addition to fecal samples. Other cell types such as buccal cells are underused.

With respect to targeted compared with untargeted approaches, the majority of carotenoid and health-related studies have been conducted in a rather non-targeted fashion (see tables), though with the exception of transcriptomics, where also many targeted approaches have been carried out. As omics-based research in this domain is still fairly recent, most approaches have thus aimed for hypothesis-building and obtaining a more general overview of the interactions of carotenoids on many bodily functions, though more targeted approaches are likely to be expected in the near future to consolidate earlier findings.

With respect to the studied carotenoids, the majority of studies has been focusing on the native, most frequently consumed carotenoids, especially lycopene, and β-carotene. However, carotenoids consumed within algae (fucoxanthin), seafood (astaxanthin), and some novel sources such as from fungi (torularhodin) have also been the subject of a number of investigations, as have been apo-carotenoids such as crocin and crocetin, which may be interesting and yet understudied carotenoids. More studies comparing and contrasting the potential differential effects of various carotenoids and metabolites are also warranted.

Regarding transcriptomics, many targeted approaches employing real-time and quantitative PCR have been carried out, and a number of interesting molecular targets, such as transcription factors, but also markers of inflammation, oxidative stress, and the immune system, have been identified to be impacted by dietary exposure to carotenoids (Table 1). Targeted approaches using DNA microarrays and RNA-seq remain somewhat in the minority, as do approaches including microRNAs and lncRNAs, something that has only scarcely been investigated in relation to carotenoid bioactivity and which may present an interesting future direction. For sure, the larger and more affordable availability of microarrays and even RNA sequencing will increase the number of studies that measure potential health-related targets of carotenoids.

With respect to proteomics, human studies still have remained the exception (Table 2), and those have been investigating the relationship of circulating carotenoids with plasma proteins and thus did not include randomized clinical trials. Overall, most studies have been using MS-based methods for protein separation and not 2D-DIGE approaches, and all of the approaches were non-targeted. Therefore, there clearly exists an area of growth in applying proteomics to human studies, especially intervention trials. Here, assay-based methods (e.g., SomaScan) may provide an affordable compromise to more classical approaches. This may perhaps avoid some of the heavy costs related to stable isotope labeling approaches (SILAN, SILAC, ICAT). A main challenge may be to detect the impact of minor abundant proteins, requiring pre-selecting the right cellular (sub-) fraction or selectively removing major abundant proteins (e.g., by immunoprecipitation).

Regarding metabolomics/lipidomics, human studies have likewise been rare but have been including both observational and interventional approaches (Table 3). Untargeted approaches are clearly dominant, as has been a certain focus on lycopene, though also novel (apo-)carotenoids (crocetin, torularhodin) have been studied, at least in animal models, for their relation to obesity aspects and against cerebral ischemia, respectively. Mostly, triglycerides and cholesterol fractions have been studied, though there appears to be room for including a broader number of lipid classes, such as prostaglandins. The main obstacles are still the high pricing of the instrumentation, as well as missing available standards or libraries to compare results with. This is especially true for lipidomics, including carotenoid analysis, which faces the double burden of often very low concentrations (nanomolar range) together with the absence of standards.

Metagenomics and metatranscriptomics of the gut microbiota have been in a clear minority related to carotenoid-focused research (Table 4), potentially because knowledge on carotenoids in microbes primarily focuses on their roles in photosynthesis, which obviously does not occur in the gut. However, because of the role of carotenoids in protecting against oxidative stress, a handful of metaomics approaches have been taken to study the effects of carotenoids within the past ten years. Of these, 16S rRNA sequencing is the most common due to the ease of library preparation and modular packages for data analysis. Unfortunately, short-read 16S sequencing can only reveal microbes at the genus level, which masks important metabolic differences between species (especially regarding secondary metabolites like carotenoids). We expect that future studies on carotenoids and the microbiome will utilize much more informative sequencing approaches, such as long-read 16S sequencing, metagenomics, and metatranscriptomics, which can reveal microbial alterations at the gene level, which is much more mechanistically informative. Indeed, the single metagenomic sequencing study available in this area revealed key impacts of a single enzyme class, an example would be phytoene dehydrogenase. These studies, combined with improved annotation of carotenoid-active enzymes more generally, should illuminate what happens to dietary carotenoids when they make their way into the gut microbiome.

Features that apparently need to be improved have been mentioned in several omics based reviews across the various omics domains. These include, in addition to technological developments, especially tools that facilitate handling a large amount of data, including statistical tools such as machine learning methods [230,231] and also biological interpretations such as more holistic pathway analyses [232,233], and more databases regarding gene-expression data including animals [234], proteins [235,236], metabolites [237,238], and the microbiome [239,240].

Future developments in the carotenoid research field will likely include broader and more affordable solutions that cover the above omics domains, as well as combinations thereof, i.e., integrated/multiomics, which surely will allow obtaining more insights into the rather complex metabolism and multiple potential target sites in the body that respond to dietary intake of these health-associated pigments. The future of carotenoid research in this respect is colorful and bright.

## Author contributions

The authors’ contributions were as follows— AE, TB, EB, HU, NC, MI, GW: conception, writing, and revision of the manuscript; AE, TB: prepared the outline for the overall manuscript, wrote the introduction and conclusions/perspectives, and adapted the individual chapters; MH: drafted the tables; NC, GW wrote the chapter on metagenomics/metatranscriptomics; EB: contributed to transcriptomics, proteomics, metagenomics chapters; HU: helped to write the proteomics chapter; and all authors: have read and approved the final content.

## Conflict of interest

The authors report no conflict of interest.

## Funding

AE received funds from the USDA NIFA AFRI Foundational and Applied Science Program (A1343 – Food and Human Health), Grant Award Number: 2022-67018-37188.
